# Adipocyte browning and resistance to obesity in mice is induced by expression of ATF3

**DOI:** 10.1038/s42003-019-0624-y

**Published:** 2019-10-24

**Authors:** Ching-Feng Cheng, Hui-Chen Ku, Jing-Jy Cheng, Shi-Wei Chao, Hsiao-Fen Li, Pei-Fang Lai, Che-Chang Chang, Ming-Jaw Don, Hsi-Hsien Chen, Heng Lin

**Affiliations:** 1 Department of Pediatrics, Taipei Tzu Chi Hospital, Buddhist Tzu Chi Medical Foundation, Taipei, Taiwan; 2 Institute of Biomedical Sciences, Academia Sinica, Taipei, Taiwan; 3 Department of Pediatrics, Tzu Chi University, Hualien, Taiwan; 4 Ph.D. Program in Biotechnology Research and Development, Taipei Medical University, Taipei, Taiwan; 5 Department of Physiology, School of Medicine, College of Medicine, Taipei Medical University, Taipei, Taiwan; 6 Ph.D. Program in Clinical Drug Discovery from Botanical Herbs, Taipei Medical, University, Taipei, Taiwan; 7 National Research Institute of Chinese Medicine, Taipei, Taiwan; 8 Department of Emergency Medicine, Buddhist Tzu Chi General Hospital, Hualien, Taiwan; 9 Division of Nephrology, Department of Internal Medicine, School of Medicine, College of Medicine, Taipei Medical University, Taipei, Taiwan; 10 Division of Nephrology, Department of Internal Medicine, Taipei Medical University Hospital, Taipei, Taiwan

**Keywords:** Drug discovery, Transcription, Obesity, Diabetes, Medical research

## Abstract

Billions of people have obesity-related metabolic syndromes such as diabetes and hyperlipidemia. Promoting the browning of white adipose tissue has been suggested as a potential strategy, but a drug still needs to be identified. Here, genetic deletion of activating transcription factor 3 (*ATF3*^*−/−*^) in mice under a high-fat diet (HFD) resulted in obesity and insulin resistance, which was abrogated by virus-mediated ATF3 restoration. ST32da, a synthetic ATF3 inducer isolated from *Salvia miltiorrhiza*, promoted ATF3 expression to downregulate adipokine genes and induce adipocyte browning by suppressing the carbohydrate-responsive element-binding protein–stearoyl-CoA desaturase-1 axis. Furthermore, ST32da increased white adipose tissue browning and reduced lipogenesis in HFD-induced obese mice. The anti-obesity efficacy of oral ST32da administration was similar to that of the clinical drug orlistat. Our study identified the ATF3 inducer ST32da as a promising therapeutic drug for treating diet-induced obesity and related metabolic disorders.

## Abstract (web summary)

Ching-Feng Cheng et al. find that expression of the transcription factor ATF3 in mice provides protection from obesity and insulin resistance under a high-fat diet. They show that expression of ATF3 can be induced by ST32da, a compound derived from the Chinese sage plant, *Salvia miltiorrhiza*.

## Introduction

The prevalence of obesity, a risk factor for type 2 diabetes, hyperlipidemia and non-alchoholic fatty liver disease, has reached epidemic proportions worldwide. Mammals have two different types of adipose tissue: white adipose tissue (WAT) and brown adipose tissue (BAT). WAT primarily stores lipids, and BAT confers anti-obesity effects by metabolizing lipids through uncoupling protein 1 (UCP1)-mediated uncoupled respiration^1,2^. In small mammals, BAT plays a key role in the thermogenic response and regulation of energy balance^3^. Moreover, BAT activation promotes energy expenditure, reduces adiposity, and protects against diet-induced obesity^4,5^. Recent studies have shown the existence of another type of adipocyte: beige or “brite” (brown in white) cells^2,6,7^. In addition, morphological and histological data have shown adipocytes with an intermediate phenotype, which may due to white-to-beige adipocyte transdifferentiation^8,9^. In this regard, strategies to combat obesity have shifted from reducing fat accumulation to promoting energy expenditure by activation of BAT and browning of WAT^10–13^.

Two transcription-factor families are key determinants of terminal adipocyte differentiation: CCAAT/enhancer-binding proteins C/EBPα, C/EBPβ, and C/EBPδ, and peroxisome proliferator-activated receptor γ (PPARγ). Previous studies have shown that activating transcription factor 3 (ATF3) can inhibit the expression of C/EBPα, which in turn can inhibit adipocyte differentiation^14^. ATF3 levels are high in the WAT of obese mice and negatively regulate adiponectin gene expression^15^. In addition, ATF3 is involved in adipocyte hypoxia-mediated mitochondrial dysfunction in obesity^16^. Although the biological functions of ATF3 have been widely studied, the specific role of ATF3 in obesity regulation and energy metabolism remains to be fully explored.

In the present study, deletion of *ATF3* in mice aggravated high-fat diet (HFD)-induced obesity and metabolic dysfunction. Furthermore, ATF3 overexpression inhibited adipogenic/lipogenic gene expression and upregulated lipolytic and browning-related gene expression, which was due to suppressing the gene expression of carbohydrate-responsive element-binding protein (*ChREBP*) and stearoyl-CoA desaturase-1 (*Scd1*). We used ATF3-specific promoter-screening approaches to explore ATF3 inducers from a modified Chinese herb single-compound library and uncovered a single compound, ST32da, a synthetic compound related to neo-tanshinlactone and isolated from *Salvia miltiorrhiza*, that showed good anti-obesity effects in mice with HFD-induced obesity. We confirmed that ATF3 is an important metabolic regulator. The ATF3-inducer ST32da may have therapeutic benefits in individuals with obesity and metabolic dysfunction.

## Results

### Obese patients had reduced ATF3 levels in different organs

**Fig. 1:**
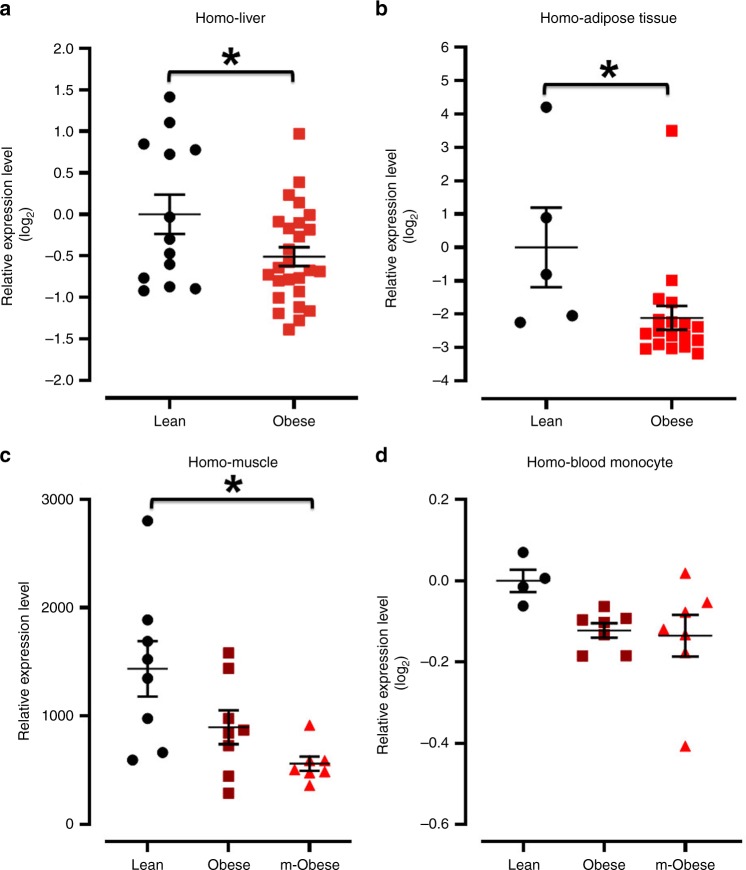
Analysis of ATF3 expression level among liver, adipose tissue, muscle and blood monocytes from lean, obese and morbidly obese patients by NCBI GEO DataSets. **a**–**d** ATF3 expression level in different organs. **a** Liver. **b** Adipose tissue. **c** Muscle. **d** Blood monocytes. For **a**, Lean (*n* = 13), Obese (*n* = 26). For **b**, Lean (*n* = 5), Obese (*n* = 18). For **c**, ControlLean (*n* = 8), Obese (*n* = 8), m-Obese (*n* = 7). For **d**, Lean (*n* = 4), Obese (*n* = 7), m-Obese (*n* = 7). Data are mean ± SEM. and *p < 0.05 compared to control group

Prior gene polymorphism study indicated that *ATF3* is associated with human obesity^17^. Furthermore, after analysis the relationship between ATF3 and obesity in human GEO DataSet Browser (https://www.ncbi.nlm.nih.gov/sites/GDSbrowser/), we characterize that the gene expression of ATF3 was lower in human liver (Fig. 1a)^18^, adipose tissue (Fig. 1b)^19^ and muscle (Fig. 1c)^20^ specimens of obese than in the lean ones, but the ATF3 expression did not differ in the blood monocytes from normal weight, mildly obese and morbidly obese subjects (Fig. 1d)^21^.

### *ATF3*^−/−^ mice conferred HFD-induced metabolic dyshomeostasis

**Fig. 2:**
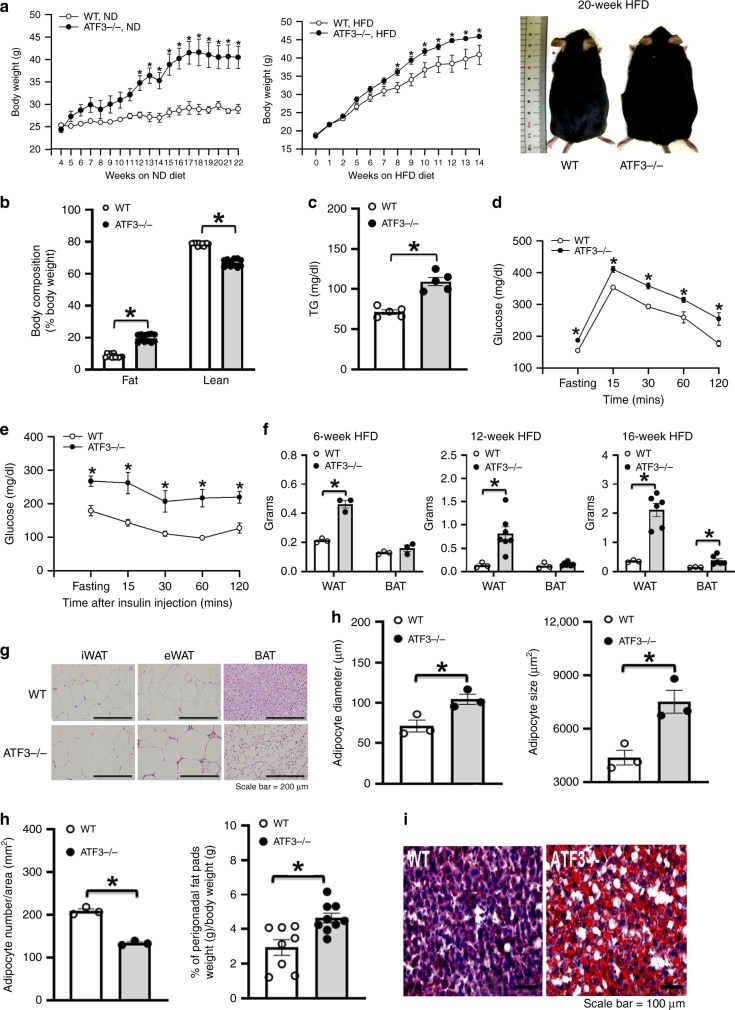
Loss of *ATF3* in mice aggravated high fat diet (HFD)-induced obesity and metabolic dysfunction. *ATF3* gene-deleted mice (*ATF3*^−/−^) and their wild-type littermates (WT) fed a normal diet (ND) or a HFD for 22 or 14 weeks, respectively. Measurement was performed after 12 weeks of HFD feeding for both groups. **a** Body weight of wild-type (WT) and *ATF3*^−/−^ mice fed a normal diet (ND) or HFD and body image. **b** Body composition of wild-type and *ATF3*^−/−^ mice. Proportion of body fat and lean mass as a percentage of their respective body weights. **c** Serum triglycerides (TG) level. **d** Glucose tolerance test. **e** Insulin tolerance test. **f** White adipose tissue (WAT) and brown adipose tissue (BAT) fat-pad weights. **g** Representative H&E staining of inguinal WAT (iWAT), epididymal WAT (eWAT), and BAT. **h** Adipocyte diameter (µm), size (μm^2^), number per area (mm^2^), and perigonadal fat pad weight per body weight (g). **i** Oil-red O staining in liver. For **a**, *n* = 3 per group (ND); wild-type, HFD (*n* = 4), *ATF3*^*−/−*^, HFD (*n* = 9). For **b**, *n* = 12 per group. For **c**, *n* = 5 per group. For **d**, wild-type (*n* = 4), *ATF3*^*−/−*^ (*n* = 5). For **e**, *n* = 3 per group. For **f**, wild-type (*n* *=* 3), *ATF3*^*−/−*^ (*n* *=* 3 in 6-week HFD; *n* *=* 7 in 12-week HFD; *n* *=* 6 in 16-week HFD). For **g**–**i**, *n* = 3 per group, except wild-type (*n* = 8), *ATF3*^*−/−*^ (*n* = 9) in % of perigonadal fat pads weight/body weight. Scale bar for images **g**: 200 µm; **i**: 100 µm. Data are mean ± SEM. and *p < 0.05 compared to wild-type

**Fig. 3:**
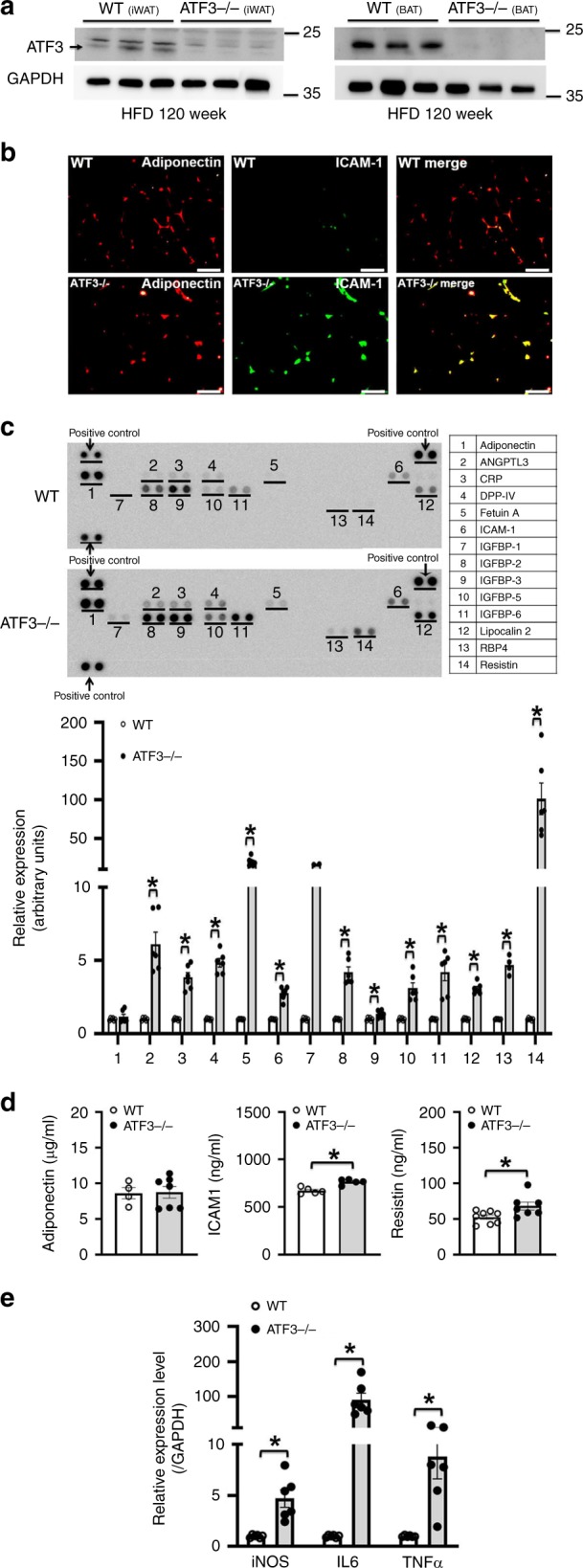
Loss of *ATF3* aggravated the expression of inflammation-related genes in HFD-induced obese mice. **a** ATF3 protein level in iWAT and BAT of wild-type and *ATF3*^−/−^ mice after HFD feeding for 12 weeks. **b** Representative immunofluorescence images of adiponectin (red IF) and ICAM-1 (green IF) in wild-type and *ATF3*^−/−^ mice. Yellow scale bar indicated the size of adipocyte tissues. **c** Serum protein levels of adipokine and inflammation-related genes in wild-type and *ATF3*^−/−^ mice after HFD feeding for 8 weeks by adipokine assays; Gel-Pro Analyzer software was used for densitometry of blots. **d** Serum protein level of adiponectin, ICAM-1 and resistin by ELISA assays in wild-type and *ATF3*^−/−^mice after HFD feeding for 8 weeks. **e** Quantified real-time PCR analysis of mRNA levels of iNOS, IL-6, and TNFα in livers of wild-type and *ATF3*^−/−^ mice. For **a**–**c**, *n* = 3 per group. For **d**, wild-type (*n* = 4 in adiponectin; *n* = 5 in ICAM1; *n* = 8 in resistin), *ATF3*^*−/−*^ (*n* = 7 in adiponectin; *n* = 5 in ICAM1; *n* = 7 in resistin). For **e**, *n* = 6 per group. Scale bar for image **b**: 50 µm. Data are mean ± SEM; *p < 0.05 compared to wild-type

To examine whether ATF3 modulates energy metabolism and body-weight changes in mice, we analyzed the metabolic syndrome and its related traits in *ATF3*-null (*ATF3*^−/−^) mice. *ATF3*^−/−^ mice, with a normal diet or HFD, showed higher body weight than their wild-type littermates (Fig. 2a). HFD-fed *ATF3*^−/−^ mice showed increased body-fat percentage, serum triglyceride (TG) levels (Fig. 2b, c), glucose intolerance and insulin resistance (Fig. 2d, e), WAT and BAT depot weights (Fig. 2f), BAT whitening (Fig. 2g, Supplementary Fig. 1a), white-adipocyte cell size, diameter and perigonadal fat-pad weight (Fig. 2g, h), and liver lipid deposition (Fig. 2i, Supplementary Fig. 1b). In addition, we found that chronic HFD (12 weeks) in mice conferred ATF3 overexpression in WAT and BAT, found only in wild-type but not *ATF3*^−/−^ mice (Fig. 3a, Supplementary Fig. 1c), implying WAT and BAT act as a stress response organ to chronic HFD. Because obesity is closely associated with a state of chronic, low-grade inflammation in adipose tissues^22^, the expression of intercellular adhesion molecule 1 (ICAM-1) but not adiponectin was higher in WAT of *ATF3*^−/−^ than wild-type mice (Fig. 3b, Supplementary Fig. 1d). Adipokines secreted from adipose tissue may have systemic effects and shift to pro-inflammation as adipose tissue expands during the development of obesity^23^. Accordingly, protein array and ELISA analysis of adipokines revealed higher serum levels of pro-inflammatory adipokines except for adiponectin from *ATF3*^−/−^ than wild-type mice (Fig. 3c, d, Supplementary Fig. 1e). Further evidence of systemic metabolic involvement, including levels of tumor necrosis factor-α (TNFα), interleukin 6 (IL-6), and inducible nitric oxide synthase (iNOS), were also higher in liver tissue of *ATF3*^−/−^ than wild-type mice by real-time PCR analysis (Fig. 3e). These results from immunofluorescence, protein array and real-time PCR analyses all showed that ATF3 may inhibit the secretion of inflammatory hormones in adipose tissue.

### ATF3 ameliorated HFD-induced metabolic dyshomeostasis

**Fig. 4:**
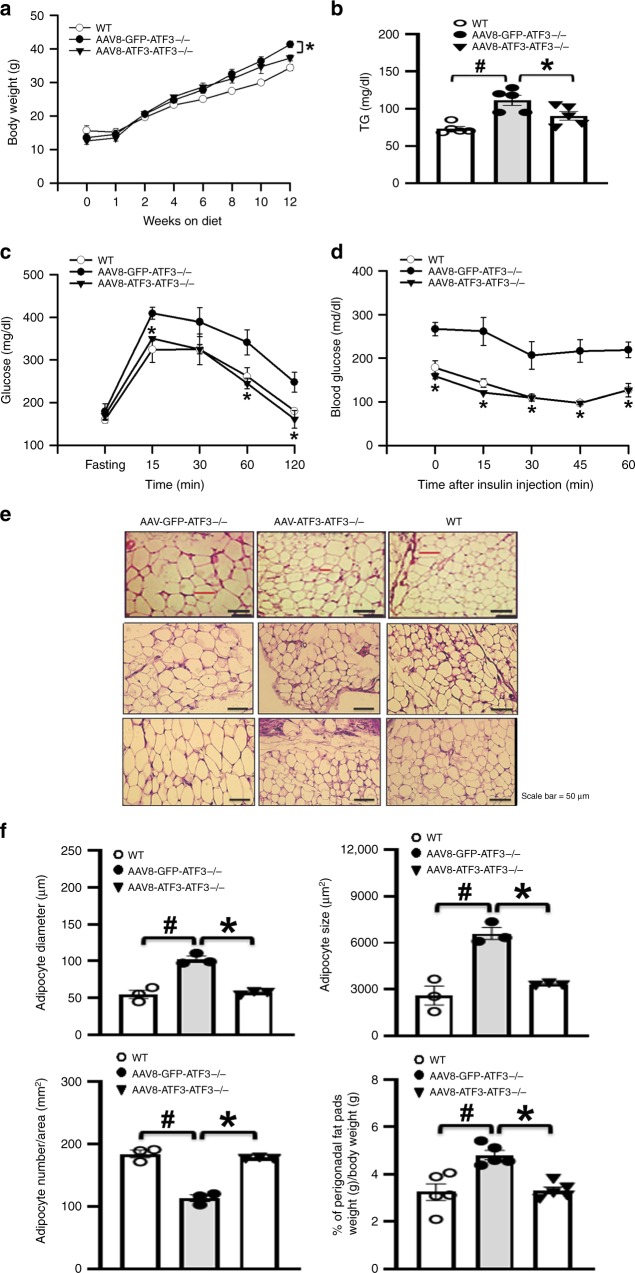
Adeno-associated virus 8 (AAV8)-mediated expression of *ATF3* reversed metabolic dysfunction in *ATF3*^−/−^ mice. Analysis of mice fed an HFD for 12 weeks: untreated wild-type mice, AAV8-GFP–treated *ATF3*^−/−^ mice (AAV8-GFP- *ATF3*^−/−^), or AAV8-*ATF3*-treated *ATF3*^−/−^ mice (AAV8-*ATF3*-*ATF3*^−/−^). **a** Body weights. **b** Serum TG level. **c** Glucose tolerance test. **d** Insulin tolerance test. **e** Representative H&E staining of epididymal WAT. **f** Adipocyte diameter (μm), size (μm^2^), number per area (mm^2^), and perigonadal fat pad weight per body weight (g). For **a**, *n* = 3 per group. For **b**, *n* = 5 per group. For **c**–**e**, *n* = 3 per group. For **f**, *n* = 3 per group, except wild-type (*n* = 5), AAV8-GFP-*ATF3*^*−/−*^ (*n* = 5), AAV8-*ATF3*-*ATF3*^*−/−*^ (*n* = 5) in % of perigonadal fat pads weight/body weight. Scale bar for image **e**: 50 µm. Data are mean ± SEM; ^#^*p* < 0.05 compared to wild-type. *p < 0.05 AAV8-GFP-*ATF3*^*−/*−^ vs. AAV8-ATF3-*ATF3*^*−/*−^

As a complementary approach to our knockout mouse studies, we performed phenotype-rescue studies in which *ATF3*^−/−^ mice received adeno-associated virus (AAV)-mediated gene transfer of *ATF3* (AAV8-*ATF3*)^24,25^. We found first that ATF3 expression in adipocytes was restored by AAV8-*ATF3* injection (Supplementary Fig. 2, Supplementary Fig. 1f). Next, 12 weeks after intravenously injecting HFD-fed *ATF3*^−/−^ mice with AAV8-*ATF3*, body weight, serum TG levels, glucose intolerance, and insulin resistance were lower than in *ATF3*^−/−^ mice injected with AAV8-GFP (Fig. 4a–d). In addition, peri-gonadal fat-pad weight per body weight, adipocyte diameter, and adipocyte size were lower with AAV8-*ATF3* than AAV8-GFP injection (Fig. 4e, f). These results suggest that ATF3 is a key regulator in HFD-induced obesity and related forms of metabolic dyshomeostasis.

### *ATF3*^−/−^ mice impaired WAT/BAT balance and thermoregulation

**Fig. 5:**
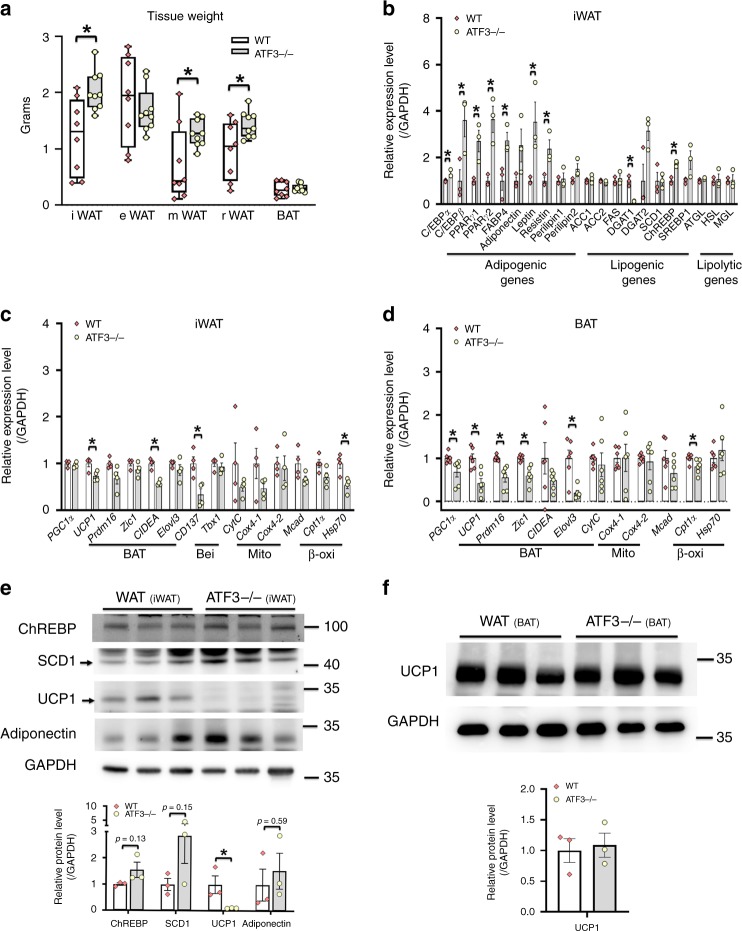
*ATF3*^−/−^ mice showed dysregulated WAT/BAT balance. Analysis of *ATF3*^−/−^ and wild-type mice after 12 weeks of HFD feeding. **a** Weights of brown adipose tissue (BAT) and white adipose tissue (WAT) in individual depots including inguinal WAT (iWAT), epididymal WAT (eWAT), mesenteric WAT (mWAT), and retroperitoneal WAT (rWAT) fat pads. **b** Analysis of gene expression of adipogenic, lipogenic, and lipolytic genes in iWAT. **c** Analysis of gene expression of brown (BAT), beige (Bei), mitochondria (Mito), and β-oxidation (β-oxi) markers in iWAT. **d** Analysis of expression of brown/mitochondria/β-oxidation markers in BAT. **e** Protein levels of ChREBP, SCD1, UCP1 and adiponectin in iWAT. **f** Protein level of UCP1 in BAT. For **a**, wild-type (*n* *=* 8), *ATF3*^*−/−*^ (*n* *=* 9). For **b**, *n* = 3 per group. For **c**, *n* = 4 per group. For **d**, *n* = 6 per group. For **e**, **f**, *n* = 3 per group. Data are mean ± SEM; *p < 0.05 compared to wild-type

**Fig. 6:**
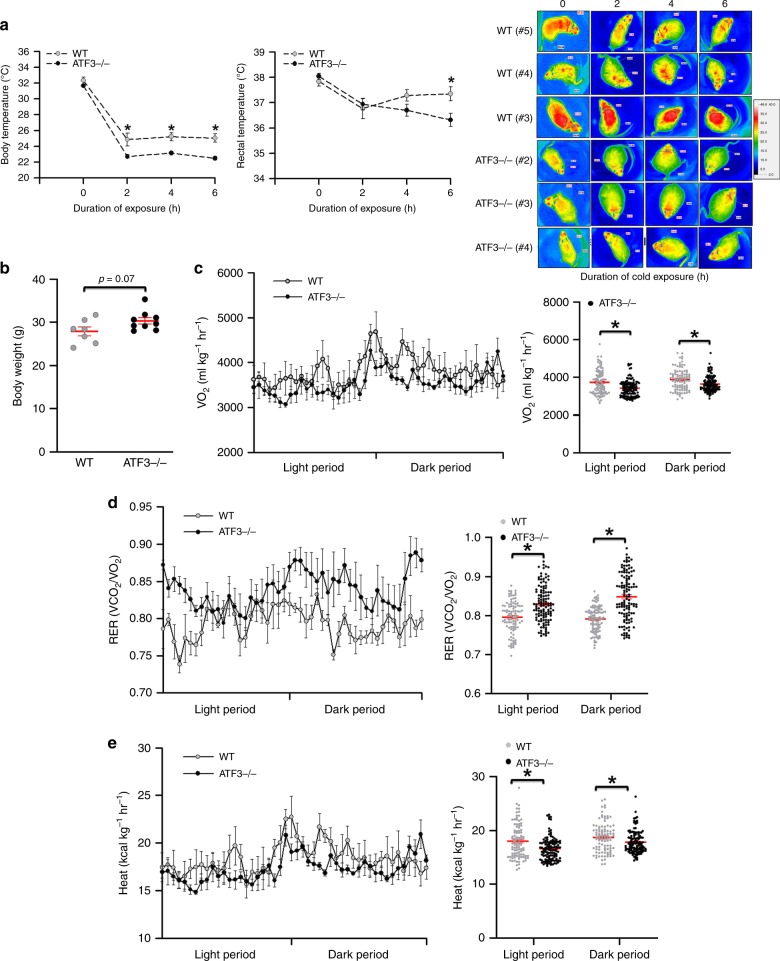
*ATF3*^−/−^ mice showed impaired energy metabolism and thermoregulation. **a** Body and rectal temperature during acute cold exposure. **b** Body weight after 9 weeks of HFD feeding. **c** Measurement of oxygen consumption levels, **d** respiratory exchange ratio (RER), and **e** energy expenditure in *ATF3*^−/−^ and wild-type mice after 9 weeks of HFD feeding. For **a**, wild-type (*n* *=* 6), *ATF3*^*−/−*^ (*n* *=* 5). For **b**, wild-type (*n* *=* 7), *ATF3*^*−/−*^ (*n* *=* 9). For **c**–**e**, wild-type (*n* *=* 4), *ATF3*^*−/−*^ (*n* *=* 5). Data are mean ± SEM; *p < 0.05 compared to wild-type

In mice, induction of heat production in BAT helps prevent fluctuations in body temperature upon cold exposure. We checked whether *ATF3*^−/−^ mice fed with a HFD affected their WAT/BAT depot distribution, energy expenditure, and thermoregulation, thus resulting in metabolic dysregulation. *ATF3*^−/−^ mice showed increased weight in inguinal WAT (iWAT), mesenteric WAT (mWAT), and retroperitoneal WAT (rWAT) white fat depots, with no difference in BAT or epididymal WAT (eWAT) (Fig. 5a). In addition, the expression of adipogenesis and lipogenesis biomarkers, including C/EBPα, c/EBPβ, PPARγ1, PPARγ2, fatty acid binding protein 4 (FABP4), resistin and ChREBP was elevated in iWAT of *ATF3*^−/−^ mice (Fig. 5b). The expression of some adipogenesis and lipogenesis biomarkers, including adiponectin, perilipin 1/2, ACC1/2, FAS, and SCD1, was not altered (Fig. 5b). In contrast, the expression of selected markers of WAT browning and BAT activation such as UCP1, CIDEA, and CD137 in iWAT (Fig. 5c) and UCP1, PGC1α, Prdm16, Zic1, and Elovl3 in BAT (Fig. 5d) was lower in *ATF3*^−/−^ than wild-type mice. Furthermore, the protein levels of ChREBP, SCD1 and adiponectin in iWAT did not differ between *ATF3*^−/−^ and wild-type mice (Fig. 5e, Supplementary Fig. 1g). Conversely, UCP1 protein was lower in iWAT (Fig. 5e, Supplementary Fig. 1g) but not BAT (Fig. 5f, Supplementary Fig. 1h) in *ATF3*^−/−^ than wild-type mice. As an alternative approach to *ATF3*^−/−^ mice, restoration of ATF3 could inhibit both ChREBP and SCD1 protein levels (Supplementary Fig. 3, Supplementary Fig. 1i). These results suggest that the loss of ATF3 in vivo affected the WAT/BAT balance and normal BAT activation. Moreover, whitening of BAT was greater for *ATF3*^−/−^ than wild-type mice (Fig. 2g), which was consistent with the downregulation of mRNA levels of BAT/beige markers in BAT of *ATF3*^−/−^ compared to wild-type mice. These results indicate that ATF3 may play an important role in promoting the white fat phenotype to brown/beige fat phenotype. Interestingly, positive correlations between ATF3 and HSL and CIDEA were observed in patients by using GEO and the GDS3679 dataset (Supplementary Fig. 4)^19^. The dysregulated balance of WAT/BAT seen in *ATF3*^−/−^ mice was also associated with impaired thermoregulation under acute cold stress (Fig. 6a). We found that, even when the body weight differences between *ATF3*^−/−^ and wild-type mice was not obvious 9 weeks after HFD feeding (Fig. 6b), the total body oxygen consumption and heat production were lower and respiratory exchange ratio was higher in *ATF3*^−/−^ than wild-type mice (Fig. 6c–e). These data implied a decrease in the use of fat (versus carbohydrate) for heat production and confirm that ATF3 ablation in mice modulated adipose tissue and mitochondria adaptive gene programming, thereby affecting the WAT/BAT balance and impairing energy expenditure and thermoregulation.

### ATF3 regulated adipocyte browning via ChREBP-SCD1 pathway

**Fig. 7:**
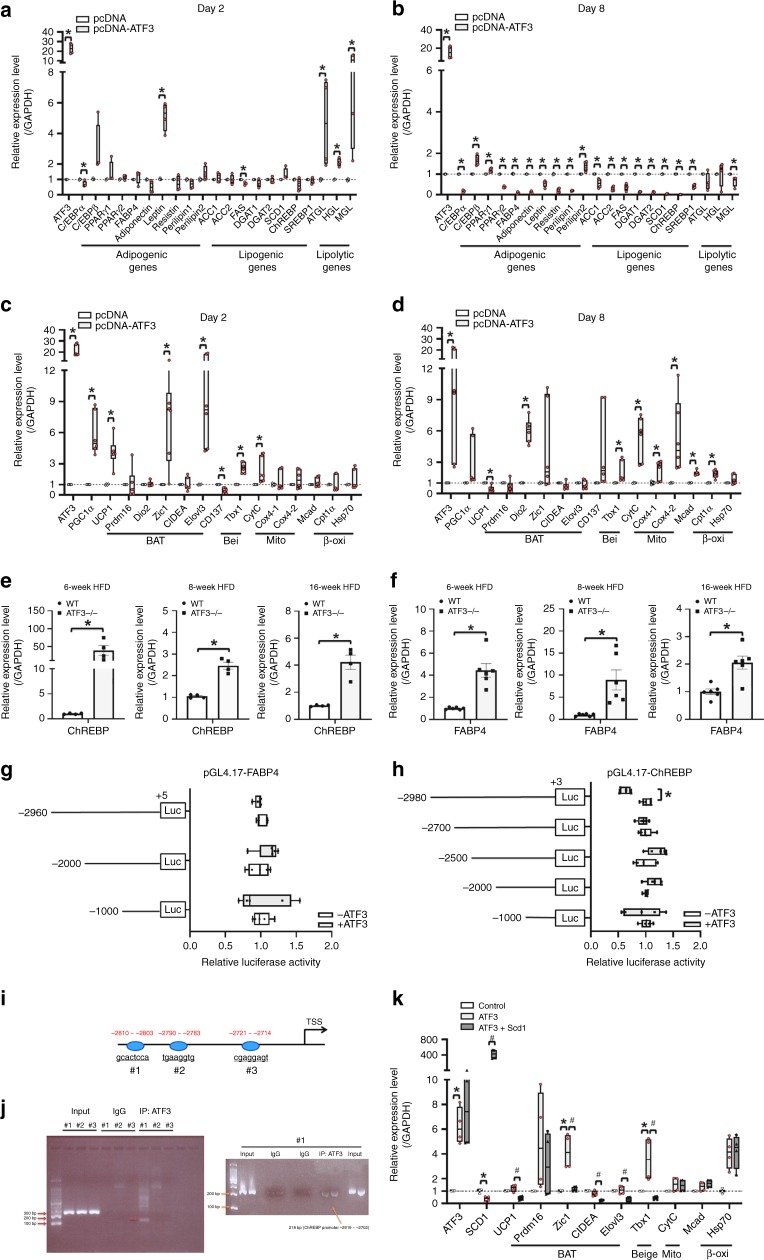
ATF3-overexpressing 3T3-L1 adipocytes showed suppression of lipogenesis/adipogenesis and activation of mitochondrial, brown or beige fat programs. **a**, **b** Real-time PCR analysis of mRNA levels of adipogenic, lipogenic, and lipolytic genes; **c**, **d** BAT, beige (Bei), mitochondria (Mito), and β-oxidation (β-oxi) genes after 2 and 8 days of differentiation in ATF3-overexpressing 3T3-L1 cells, normalized to GAPDH and relative to pcDNA control. **e**, **f** The expression level of ChREBPand FABP4 in iWAT of wild-type and *ATF3*^−/−^ mice after 6, 8, and 16 weeks of HFD feeding. **f** The expression level of FABP4 in iWAT of wild-type and *ATF3*^−/−^ mice after 6, 8, and 16 weeks of HFD feeding. **g** *FABP4* promoter activity measured with or without overexpression of ATF3 in 3T3-L1 pre-adipocytes. **h** Overexpression of ATF3 repressed the *ChREBP* promoter activity of the p (−2980)/Luc reporter but not other reporters in 3T3-L1 pre-adipocytes. **i** The sequence of 3 potential binding sites for ATF3 in *ChREBP* promoter, including region #1 (–2810/–2803), region #2 (−2790/−2783) and region #3 (−2721/−2714) of the *ChREBP* locus. **j** Chromatin immunoprecipitation (ChIP) experiments with ATF3-specific antibody and primers to amplify region #1, region #2 and region #3 of the *ChREBP* locus, which contains one predicted ATF/CRE binding site in 3T3-L1 preadipocytes. **k** Real-time PCR analysis of gene levels of brown (BAT), mitochondrial (Mi), beige (Bei), and β-oxidation (β-oxi) genes in ATF3-overexpressing 3T3-L1 pre-adipocyte stable clone with or without *Scd1* transfection. For **a**, **b**, *n* = 4 per group. For **c**, **d**, pc-DNA (*n* = 4), pc-DNA-*ATF3* (*n* = 6). For **e**, *n* = 4 per group. For **f**, *n* = 6 per group. For **g**, **h**, *n* = 5 per group. For **j**, *n* = 3 per group. For **k**, control (*n* = 3), ATF3 (*n* = 4), ATF3 + SCD1 (*n* = 4). Data are mean ± SEM; *p < 0.05 compared to control. ^**#**^*p* < 0.05 ATF3 vs. ATF3 + SCD1

To clarify the molecular mechanism underlying the regulation of lipogenesis/lipolysis and energy expenditure in white adipocytes by ATF3, we stably overexpressed *ATF3* in 3T3-Ll cells. Overexpression of *ATF3* decreased (>80%) oil droplet deposition in 3T3-Ll cells after 8 days of differentiation (Supplementary Fig. 5), so normal adipogenesis was suppressed. Further examination of markers related to adipogenesis and lipogenesis, including PPARγ, c/EBPα, ACC1/2, ChREBP, and SCD1, showed reduced levels in ATF3-overexpressing cells^26^ (Fig. 7a, b). By contrast, the expression of genes involved in BAT/beige fat programs and β-oxidation, such as UCP1, PGC1α, Cpt1α and Mcad, was upregulated in ATF3-overexpressing cells (Fig. 7c, d). These data were consistent with our in vivo results that expression of adipogenesis and lipogenesis biomarkers was oppositely elevated in iWAT of *ATF3*^−/−^ mice (Fig. 5b).

ChREBP is a glucose-responsive transcription factor that drives de novo lipogenesis and increases adiposity and potentially worsens insulin resistance^27,28^. Therefore, we checked whether ChREBP is involved in the ATF3-dependent lipogenesis/lipolysis balance. The expressions of ChREBP (Fig. 7e) and FABP4 (Fig. 7f) were increased in *ATF3*^−/−^ mice after a HFD for 6–16 weeks. To examine whether ATF3 could regulate ChREBP or FABP4 directly, we analyzed the proximal promoter regions of *ChREBP* and *FABP4*. Sequential 5′-deleted luciferase reporter constructs with different lengths of *ChREBP* and *FABP4* promoter regions were created and expressed with and without ATF3 in 3T3-L1 pre-adipocytes. We found that *FABP4* promoter activity was not repressed by ATF3 (Fig. 7g). Only the −2980 construct of *ChREBP* promoter was repressed by ATF3 (Fig. 7h), which suggested that the *ChREBP* promoter (from −2980 to −2700) is involved in the ATF3-dependent regulation of ChREBP. Furthermore, we identified three potential ATF3-binding sites (Fig. 7i). To confirm this finding, we used chromatin immunoprecipitation assay to examine whether ATF3 could bind to its potential binding sites upstream of the *ChREBP* promoter. ATF3 bound to site 1 but not sites 2 and 3 (Fig. 7j).

ChREBP can promote lipogenesis by directly regulating SCD1^29^, and mice with *Scd1* deletion show increased white adipocyte browning^30^. To check whether ATF3 activates white adipocyte browning by suppressing ChREBP–SCD1 signaling, we overexpressed SCD1 in ATF3-overexpressing 3T3-L1 cells and examined the expression of BAT/beige markers. SCD1 overexpression reduced the upregulation of BAT/beige markers, including UCP1, Zic1, CIDEA, and Tbx1, in ATF3-overexpressing 3T3-L1 cells (Fig. 7k). Thus, ATF3 can suppress adipocyte adipogenesis and lipogenesis while activating white adipocyte transdifferentiation by inhibiting ChREBP and SCD1.

### Identification of the small-molecule ATF3-inducer ST32da

**Fig. 8:**
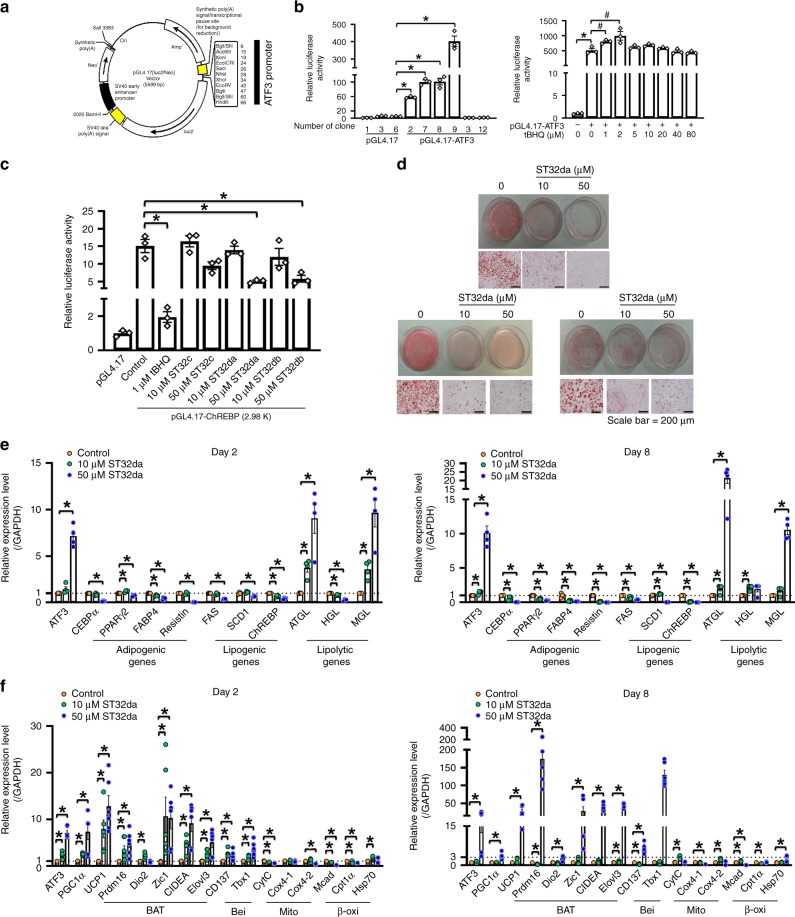
Identification of ATF3 inducers and their functional assays. **a** Construction map of *ATF3* promoter in pGL4.17 plasmid containing luciferase cassette. **b** Luciferase activity of stable clones of 3T3-L1 pre-adipocytes expressing pGL4.17-*ATF3*, with tBHQ as a positive control. **c** Luciferase activity measured in 3T3-L1 pre-adipocytes transfected with pGL4.17-*ChREBP* (p (−2980)/Luc reporter), then treated with ST32da or ST32db or ST32c. **d** Oil-red O staining in differentiated 3T3-L1 adipocytes with and without the ATF3 inducer ST32da (10 and 50 µM) for 8 days. **e** Real-time PCR analysis of mRNA levels of adipogenic, lipogenic, and lipolytic genes; **f** BAT, beige (Bei), mitochondria (Mito) and β-oxidation (β-oxi) genes with 2 and 8 days of ST32da treatment during 3T3-L1 differentiation normalized to GAPDH and relative to control. For **b**–**d**, *n* = 3 per group. For **e**, *n* = 4 per group. For **f**, *n* = 6 per group. Data are mean ± SEM; *p < 0.05 compared to control. ^**#**^*p* < 0.05 com*p*ared to pGL4.17-*ATF3*

Overexpression of ATF3 decreased (>80%) oil droplet deposition, reduced the expression of adipogenic/lipogenic markers, and increased that of lipolytic markers in 3T3-Ll cells (Fig. 7a, b and Supplementary Fig. 5). These findings suggested that upregulation of ATF3 could be effective in treating or preventing obesity. We subcloned the *ATF3* promoter into the pGL4.17-Luc luciferase reporter vector and selected stable clones overexpressing pGL4.17-*ATF3* in 3T3-L1 pre-adipocytes (Fig. 8a, b). We then screened more than 400 small molecules from modified Chinese herbs single compound library at the National Research Institute of Chinese Medicine, Ministry of Health and Welfare and uncovered a single compound by using this stable clone with pGL4-*ATF3* overexpression. tBHQ, a known ATF3 inducer, was a positive control. We found 19 compounds that increased *ATF3* promoter activity and 44 that decreased it as compared with the tBHQ-treated positive control (Supplementary Table 1). Compound ST32da showed the highest luciferase activity and was chosen for further analysis. The chemical structure and purity of ST32da were characterized by mass spectrophotometry (Supplementary Fig. 6). Because we found that ATF3 inhibited *ChREBP* promoter activity (Fig. 7h, j), we attempted to clarify whether ST32da would produce similar effects. ST32da dose-dependently reduced *ChREBP* promoter activity within the p (−2980)/Luc reporter (Fig. 8c). Furthermore, ST32da dose-dependently reduced lipid accumulation in 3T3-L1 adipocytes (Fig. 8d). ST32da-treated 3T3-L1 cells showed increased ATF3 expression and high levels of lipolytic markers such as ATGL and MGL but downregulated expression of adipogenesis- and lipogenesis-related genes (Fig. 8e). In addition, ST32da-treated cells showed increased expression of BAT, beige, β-oxidation, and mitochondrial gene markers (Fig. 8f). Therefore, the effects of ST32da were similar to those of ATF3 overexpression in vitro.

### ST32da ameliorated HFD-induced metabolic dyshomeostasis

**Fig. 9:**
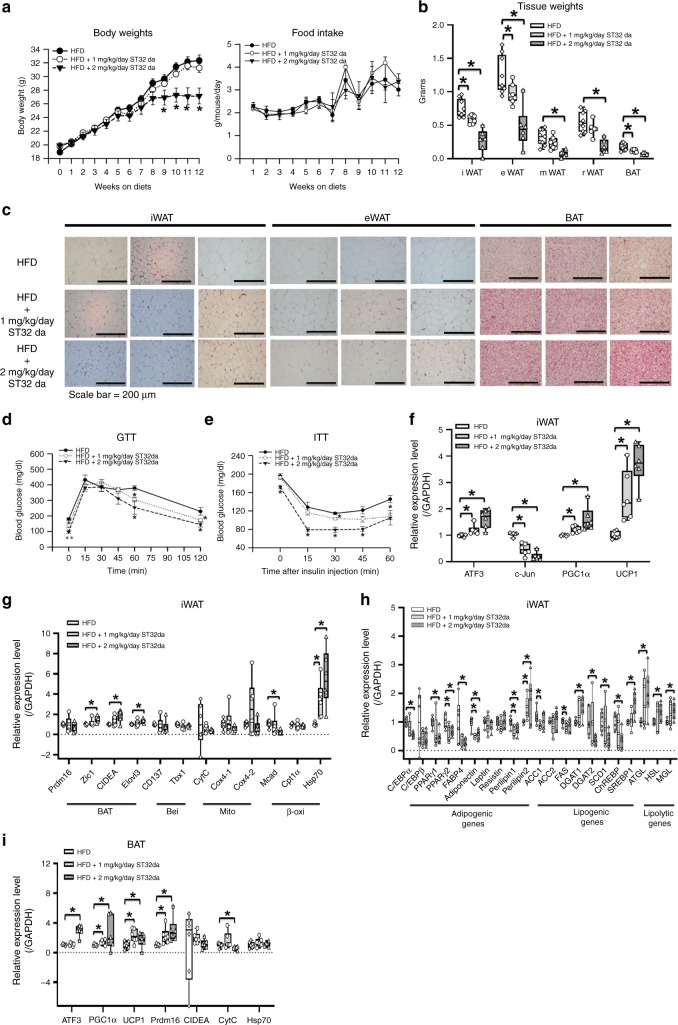
ATF3 inducer, ST32da, protects against HFD-induced obesity and metabolic dysfunction by promoting browning in vivo. Analysis of wild-type mice fed a HFD for 12 weeks with or without i.p. ST32da 1 or 2 mg kg^−1^ per day. **a** Body weight and food intake. **b** Change in adipose tissue depot weight in BAT and WAT. **c** H&E staining of inguinal WAT, epididymal WAT, and BAT fat depots. **d** Glucose tolerance test (GTT). **e** Insulin tolerance test (ITT). **f** Real-time PCR analysis of mRNA levels of ATF3, c-Jun, PGC-1α and UCP1. **g** Real-time PCR analysis of mRNA levels of brown (BAT) and beige (Bei), mitochondria (Mito), and β-oxidation (β-oxi) genes in iWAT; **h** adipogenic, lipogenic, and lipolytic genes in iWAT; and **i** brown fat programs in BAT. For **a**, HFD (*n* *=* 10), HFD + 1 mg kg^−1^ per day ST32da (*n* *=* 8), HFD + 2 mg kg^−1^ per day ST32da (*n* *=* 8). For **b**, HFD (*n* *=* 9), HFD + 1 mg kg^−1^ per day ST32da (*n* *=* 7), HFD + 2 mg kg^−1^ per day ST32da (*n* *=* 6). For **c**, *n* = 3 per group. For **d**, **e**, HFD (*n* *=* 4), HFD + 1 mg kg^−1^ per day ST32da (*n* *=* 5), HFD + 2 mg kg^−1^ per day ST32da (*n* *=* 4). For **f**, **g**, *n* = 6 per group. For **h**, HFD (*n* *=* 6), HFD + 1 mg kg^−1^ per day ST32da (*n* *=* 5), HFD + 2 mg kg^−1^ per day ST32da (*n* *=* 6). For **i**, *n* = 6 per group. Data are mean ± SEM; *p < 0.05 compared to HFD group

Having identified ST32da as the most effective ATF3 inducer, we then tested whether ST32da could influence the lipogenesis/lipolysis balance or WAT browning in vivo. HFD-fed obese mice received a daily intraperitoneal (i.p.) administration of ST32da, and food intake and body weight were measured weekly. The mice received ST32da at two different dosages (1 and 2 mg kg^−1^ per day), only higher dose (2 mg kg^−1^ per day) leading to a reduced body weight as compared with non-treated HFD-fed mice but with no significant difference in food intake (Fig. 9a).

Because *ATF3*^−/−^ mice showed increased WAT (Fig. 2f), we then examined whether the role of ST32da in mitigating the weight gain was mediated by reducing WAT weight. The weight of iWAT, eWAT, mWAT, rWAT, and BAT was dose-dependently reduced in ST32da-treated mice after 12 weeks of treatment (Fig. 9b). Different adipose depots, including iWAT, eWAT, and BAT, showed decreases in adipocyte size and diameter after ST32da treatment (Fig. 9c). In addition, ST32da enhanced the glucose tolerance and insulin sensitivity of HFD-fed obese mice (Fig. 9d, e) while decreasing serum TG and creatinine levels (Supplementary Fig. 7a). However, serum BUN levels, liver function and weight, and cardiac/renal histology remained unchanged (Supplementary Fig. 7a–d).

Overexpression of ATF3 induced transdifferentiation of white adipocytes to beige/brown adipocytes in vitro (Fig. 7c, d). Similarly, ST32da treatment increased BAT/beige-related gene expression and decreased adipogenesis/lipogenesis gene expression in the iWAT of HFD-fed mice (Fig. 9f–h). Furthermore, the expression of BAT- and beige-related genes was enhanced by ST32da in BAT (Fig. 9i). To further support our hypothesis that ST32da exerts its beneficial effect through ATF3 signaling, we treated *ATF3*^−/−^ mice with ST32da. ST32da treatment did not reduce the body weight of HFD-fed *ATF3*^−/−^ mice (Supplementary Fig. 8a). Food intake was not affected in ST32da-treated *ATF3*^−/−^ mice (Supplementary Fig. 8b). The beneficial effect of ST32da in ameliorating the metabolic disorder and in reducing the depot weight of iWAT, eWAT, mWAT, rWAT, and BAT was lost in these HFD-fed *ATF3*^−/−^ mice (Supplementary Fig. 8c–e). In addition, the inhibitory effect of ST32da on SCD1 and ChREBP expression also vanished in these HFD-fed *ATF3*^−/−^ mice (Supplementary Fig. 8f), and ST32da treatment appeared to have no effect on liver weight, adipocyte size and number in iWAT (Supplementary Fig. 8g, h) and serum adiponectin levels (Supplementary Fig. 9). Therefore, ST32da may exert its beneficial effects specifically via ATF3 activation and effectively protect against obesity in addition to lowering metabolic dysregulation in HFD-fed obese mice.

**Fig. 10:**
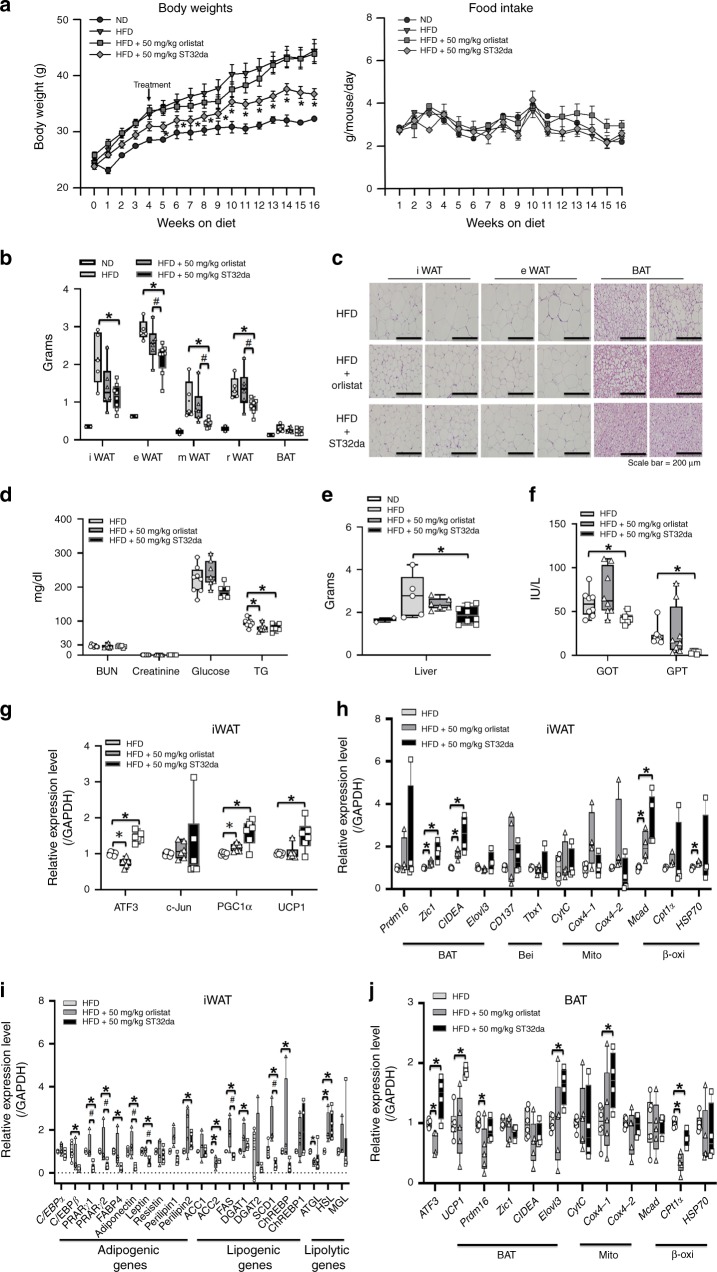
Oral administration of ATF3 inducer, ST32da, is effective in preventing HFD-induced obesity. Analysis of wild-type mice fed a HFD for 16 weeks, either without or treated with oral orlistat (50 mg kg^−1^, three times per week) or oral ST32da (50 mg kg^−1^, three times per week). **a** Body weights and food intake. **b** Variation of adipose tissue depot weight in BAT and WAT. **c** H&E staining of inguinal WAT, epididymal WAT, and BAT fat depots. **d** Serum parameters. **e** Liver weight. **f** Liver function. **g** Real-time PCR analysis of mRNA levels of ATF3, c-Jun, PGC-1α and UCP1 in iWAT; **h** brown (BAT), beige (Bei), mitochondria (Mito), and β-oxidation (β-oxi) genes in iWAT; **i** adipogenic, lipogenic, and lipolytic genes in iWAT; **j** brown/mitochondria/β-oxidation markers in BAT. For **a**, ND (*n* = 2), HFD (*n* = 7), HFD + 50 mg kg^−1^ Orlistat (*n* = 7), HFD + 50 mg kg^−1^ ST32da (*n* = 7). For **b**, ND (*n* = 2), HFD (*n* = 5), HFD + 50 mg kg^−1^ Orlistat (*n* = 6), HFD + 50 mg kg^−1^ ST32da (*n* = 8). For **c**, *n* = 3 per group. For **d**, HFD (*n* = 8), HFD + 50 mg kg^−1^ Orlistat (*n* = 7), HFD + 50 mg kg^−1^ ST32da (*n* = 6). For **e**, ND (*n* = 2), HFD (*n* = 5), HFD + 50 mg kg^−1^ Orlistat (*n* = 6), HFD + 50 mg kg^−1^ ST32da (*n* = 8). For **f**, HFD (*n* = 8), HFD + 50 mg kg^−1^ Orlistat (*n* = 8), HFD + 50 mg kg^−1^ ST32da (*n* = 6). For **g**, *n* = 6 per group. For **h**, *n* = 4 per group. For **i**, HFD (*n* = 4), HFD + 50 mg kg^−1^ Orlistat (*n* = 4), HFD + 50 mg kg^−1^ ST32da (*n* = 5). For **j**, HFD (*n* = 6), HFD + 50 mg kg^−1^ Orlistat (*n* = 5), HFD + 50 mg kg^−1^ ST32da (*n* = 4). Data are mean ± SEM; *p < 0.05 compared to HFD; ^#^*p* < 0.05 HFD + Orlistat vs. HFD + ST32da. GOT, glutamic oxaloacetic transaminase; GPT, glutamate pyruvate transaminase

Oral ST32da administration conferred anti-obesity effects similar to those of orlistat, an FDA-approved oral anti-obesity drug. In general, oral drug therapy is more convenient than intravenous injection in terms of treating obesity and obesity-related chronic disease such as type 2 diabetes. Therefore, we checked whether oral administration of ST32da to HFD-fed obese mice was beneficial and compared the results with those of orlistat (a US FDA approved anti-obesity drug). ST32da and orlistat were administered orally at 50 mg kg^−1^ to HFD-fed mice three times per week for 12 weeks. Similar to our results with i.p. ST32da treatment, oral ST32da treatment ameliorated HFD-induced obesity without affecting food intake (Fig. 10a). The decrease in WAT depot weight in ST32da-treated mice was similar to the effect of orlistat on iWAT and better than the effect on eWAT, mWAT and rWAT (Fig. 10b). Along with fat mass reduction, size of adipocytes was decreased in both WAT and BAT (Fig. 10c, Supplementary Fig. 1j). Oral ST32da decreased TG level and liver weight but did not change the biochemical profiles of renal function and serum glucose concentration as compared with orlistat and HFD only (Fig. 10d, e). Oral ST32da also lowered glutamic oxaloacetic transaminase (GOT) and glutamate pyruvate transaminase (GPT) levels as compared with orlistat and HFD only (Fig. 10f).

Similar to i.p. administration of ST32da, oral ST32da activated browning-related gene expression in both iWAT (Fig. 10g, h) and BAT (Fig. 10j), both better than oral orlistat administration and suppressed adipogenesis/lipogenesis-related gene expression in iWAT (Fig. 10i), These data confirm that oral ST32da treatment could reduce body weight and WAT fat mass to a degree similar to that with orlistat.

## Discussion

Previous studies of ATF3 mainly emphasized the diabetic syndrome and glucose homeostasis. In type 2 diabetes mellitus, ATF3 deficiency reduced serum insulin levels via reduced β-cell function^31^, and adenovirus-mediated ATF3 overexpression increased glucagon mRNA levels in αTC-1.6 cells^32^. Our results were consistent with the role of ATF3 in maintaining serum glucose homeostasis shown in these reports (Figs. 2d, e, 4c, d). In liver, ATF3 also mediates the inhibitory effects of ethanol on hepatic gluconeogenesis^33^. However, ATF3 regulation of the change in WAT and BAT in HFD-induced obesity and related metabolic disorders has not been investigated before. Here, we demonstrated that loss of ATF3 in vivo aggravated HFD-induced obesity and metabolic dysfunction in mice, with increased TG level, insulin resistance, and hepatic steatosis, along with loss of normal thermoregulation under cold stress and decreased energy expenditure. We then showed that inducing ATF3 has substantial metabolic benefits, and an ATF3 inducer, ST32da, could inhibit adipocyte lipogenesis/adipogenesis, enhance UCP1 expression, and promote white adipocyte browning in vitro, in addition to inhibiting obesity and restoring normal insulin sensitivity in HFD-fed obese mice. ATF3 plays a key role in metabolic regulation by directly suppressing the lipogenic gene *ChREBP* and enhancing adipocyte browning by inhibiting the ChREBP–SCD1 axis. Our findings confirm ATF3 inducers as promising drug candidates in treating and preventing obesity and metabolic dysfunction.

Increased visceral fat is well associated with increased metabolic disorder and morbidity and mortality from coronary heart disease, cancer, and diabetes^34^. Moreover, accumulation of visceral WAT owing to obesity may result in a stressed and dysfunctional state that leads to the release of pro-inflammatory factors such as IL-6 and TNFα, resulting in major complications such as metabolic syndrome, hepatic steatosis, neointima formation, and atherosclerosis^35,36^. Previous studies identified the anti-inflammatory, anti-apoptotic, and protective effects of ATF3 in various tissues under stress, including the brain, kidneys^25^, heart^24^, lungs^37^, and blood vessels^38,39^. ATF3 may have beneficial properties in terms of inhibiting obesity-induced cytokines in vivo by targeting muscle, adipocytes, or other organs. However, ATF3 was not found in skeletal muscle of HFD-fed obese animals^31^, and macrophage-specific ATF3-overexpressing transgenic mice did not show suppressed HFD-induced obesity^40^. Therefore, we hypothesized that ATF3 may regulate metabolic homeostasis in vivo by targeting adipocytes. Our data support this hypothesis that ATF3 regulates expression adipogenesis/lipogenesis genes in the models of ATF3-overexpressing pre-adipocytes and *ATF3*^*–/–*^ mice (Figs. 5 and 7, Supplementary Fig. 5). In addition, an ATF3 inducer, ST32da, suppressed HFD-induced obesity in mice (Figs. 9 and 10). Jang et al. also indicated that ATF3 interacts with PPARγ and represses PPARγ-mediated transactivation in white adipocytes^41^. Our data also showed that ATF3 can promote white-to-brown adipocyte transdifferentiation to increase energy expenditure and reduce WAT depots, thus mitigating obesity.

The *ATF3*^−/−^ mice fed a HFD in our study showed obesity with increased WAT weight in both subcutaneous (iWAT) and visceral depots (mWAT and rWAT) (Fig. 5a) and increased expression of many adipogenic/lipogenic genes in iWAT (Fig. 5b) In addition, the expression of browning and mitochondrial genes was reduced in the iWAT and BAT of obese *ATF3*^−/−^ mice (Fig. 5c, d), likely because of the reduced transdifferentiation of WAT to BAT; therefore, *ATF3* ^−/−^ mice showed a loss of normal thermoregulation with low body temperature under acute cold stress (Fig. 6a). In humans, the amount of BAT is also inversely proportional to body mass index and age^42^ and is barely detectable in obese patients as compared with normal-weight individuals of the same age^43^. Therefore, activating the limited BAT reserves that already exist in obese patients may be of limited therapeutic value. We speculated that ATF3 can induce WAT-to-BAT transdifferentiation and further increase BAT reserves. Our in vitro studies showed that ATF3 can induce UCP1, an adipocyte-browning indicator, and increase brown/beige-related gene expression during the early stage of 3T3-L1 cell differentiation (day 2), while repressing adipogenic/lipogenic gene expression during the late stage (day 8) (Fig. 7a–d), thereby reducing oil droplet formation (Supplementary Fig. 5b). Efficiently promoting white-to-brown transdifferentiation of adipocytes—a currently neglected topic in anti-obesity drug research—should be a primary area of future investigation.

Our in vitro data showed ATF3-overexpressing 3T3-L1 adipocytes with inhibited adipogenic/lipogenic gene expression in general (except C/EBPβ), whereas the expression of lipolytic genes on days 2 and 8 of differentiation was upregulated (Fig. 7a, b). The biphasic regulation of lipogenic or lipolytic pathways may be due to ATF3 regulating cell signalling in the context dependent manners. ATF3 homodimers have been found to act as transcriptional repressors in adipogenic/lipogenic regulation^14,44^; however, whether the ATF3 heterodimeric complex with c-Jun (or JunD) acts as a transcriptional activator of lipolytic pathways is still unclear. We found c-Jun expression reduced after i.p. administration of ST32da to mice (Fig. 9f) but no difference in c-Jun expression after oral ST32da treatment (Fig. 10g). In addition, ST32da had a similar effect in promoting lipolysis and repressing lipogenesis in vitro (Fig. 8e). ST32da also suppressed adipogenic/lipogenic gene expression and upregulated lipolytic gene expression (Fig. 9h) in adipose tissue as well as reduced WAT depots in both subcutaneous WAT (iWAT) and visceral WAT (eWAT, mWAT, and rWAT) in HFD-fed obese mice (Fig. 9b). Increased lipolysis in adipocytes may promote free-fatty acid release and worsen lipid accumulation in insulin-sensitive organs such as the liver and muscle, thus impairing insulin sensitivity^45^. However, this was not found with our ATF3 inducer in vivo. In our case, increased lipolysis was accompanied by WAT browning. BAT is an efficient modulator of triglyceridemia and considered a major plasma lipid-clearing organ in rodents^46^. Therefore, WAT browning can induce lipid clearance and promote UCP1-related mitochondrial and β-oxidation gene activation, thus lowering serum TG levels (Fig. 10d and Supplementary Fig. 7a) while increasing insulin sensitivity. Therefore, ATF3 inducers can induce insulin sensitivity and enhance body-weight loss by promoting a positive balance between both lipolysis/lipogenesis and BAT/WAT in adipocytes.

ChREBP deficiency reduced lipogenesis and glycolysis in C57BL/6J mice^47^. Recent studies showed that patients who take anti-diabetic or anti-lipid drugs, including metformin, atorvastatin, DHA/EPA, and 3-hydroxybutyrate, show reduced serum ChREBP levels^48^. Such drugs may exert their glucose- and lipid-lowering effect by modulating ChREBP trans-activity. Although ChREBP has been reported to link lipogenesis to insulin sensitivity in adipocytes^49^, whether ChREBP suppression would be beneficial in treating obesity is unclear. The *ATF3*^−/−^ mice we studied showed increased gene expression of ChREBP in iWAT after a HFD for 6 weeks (Fig. 7e). These adipogenic/lipogenic gene levels were markedly suppressed in ATF3-overexpressing 3T3-L1 cells (Fig. 7a, b). Our in vitro promoter assay and ChIP assay further confirmed that ATF3 regulates ChREBP by directly binding to the promotor region and suppressing ChREBP expression.

Overexpression of ChREBP induces SCD1, the enzyme responsible for the conversion of saturated fatty acids to monounsaturated fatty acids^29^. Although *Scd1* depletion can upregulate basal thermogenesis, thereby resulting in epididymal WAT browning^30,50^, *Scd1* deletion in liver and/or adipose tissue alone is insufficient to protect mice against HFD-induced obesity^51^. Our results showed that ATF3 could suppress SCD1 expression (Figs. 7b and 9h). However, overexpression of SCD1 can inhibit ATF3-induced BAT/beige-related gene expression (Fig. 7k), so SCD1 may act downstream of the ATF3-ChREBP repression signalling, thus confirming that ATF3 promotes WAT browning and enhances energy expenditure by repressing the ChREBP–SCD1 axis.

Over the past few years, several molecules have been tested and marketed as anti-obesity drugs. They are of three classes: centrally acting medications impairing dietary intake (serotonergic drugs) and increasing proopiomelanocortin neuron activity; medications that act peripherally to impair dietary absorption (orlistat, which inhibits lipase); and medications that increase energy expenditure, whose effect is mediated by the central nervous system (mirabegron: beta 3 adrenergic receptor agonist). The ideal way may help increase energy expenditure but only if this effect can be achieved via a direct effect on peripheral tissues without involving the central nervous system. Therefore, developing a drug that increases even minor amounts of functional BAT in the human adipose tissue could be a new, valuable approach to treating and preventing obesity and its metabolic complications.

With our *ATF3*-specific promoter screening approaches, we isolated 19 compounds that upregulate ATF3 expression (Supplementary Table 1). In addition to ST32da, other compounds, such as ST32db and ST32c, inhibited *ChREBP* promoter activity (Fig. 8c). ST32da was given to the HFD-fed obese mice i.p. or orally. Both intraperitoneal and oral delivery of ST32da could reduce adipose depot weights in iWAT, mWAT, rWAT and eWAT (Figs. 9b and 10b). Because eWAT is the main visceral depot in mice and is more susceptible to developing chronic inflammation than mWAT or iWAT^52^, our results indicate that both i.p. and oral ST32da have similar anti-obesity effect on visceral WAT reduction. On comparing the results of oral ST32da and orlistat administration, we found that oral ST32da administration had a better anti-obesity effect than orlistat with prolonged HFD (more than 12 weeks) (Fig. 10a). In addition, suppression of adipogenic/lipogenic gene expression, WAT browning, and BAT activation were greater with oral ST32da than orlistat treatment (Fig. 10g–j). Oral ST32da treatment also lowered GOT/GPT serum levels most efficiently, so oral ST32da administration is safe and does not affect the liver or renal function (Fig. 10d–f). A recent study showed that sulfuretin, a known phytochemical ATF3 inducer, could also counteract weight gain and improve glucose tolerance in HFD fed mice^53^. Our drug, ST32da, similar to sulfuretin, had the specificity of ATF3 induction (Supplementary Fig. 8). However, ST32da and sulfuretin are distinctive chemical entities, both possess completely different structures. Therefore, whether ST32da may also activate BMP, mTOR, MAPK and Wnt/β-catenin signaling pathways like sulfuretin^54^ requires further investigation. Finally, ATF3 is an adaptive response transcription factor widely expressed in various organs^24,25,37–39^. In our study, we used traditional whole-body–knockout mice. Whether ATF3 adipocyte-specific knockout in mice receiving ST32da will have the same effect as in *ATF3*-knockout mice requires further exploration.

Interactions between secretory factors from adipose tissues (including adipokines and batokines) and the nervous system (including central nervous system, innervating adipose tissues) play key roles in maintaining energy metabolism and promoting survival in response to metabolic challenges^55^. Also, the hypothalamus plays important roles in regulating brown fat activity by regulating the sympathetic nervous system activity^56^. In pancreas- and hypothalamus-specific *ATF3* knockout (PHT-*ATF3*-KO) mice, ATF3 played an important role in the control of glucose and energy metabolism by regulating agouti-related protein (Agrp), which increases food intake and reduces energy expenditure^57^. Thus, hypothalamic ATF3 is involved in adjusting glucose and energy metabolism by regulating the sympathetic nervous system activity in mice. However, i.p. or oral treatment with ST32da in mice could not increase thermoregulation under acute cold stress, which suggests that ST32da may not pass through the blood–brain barrier.

Strategies to enhance the fat-burning power of BAT in human adipose tissue are important in treating obesity and currently comprise a major research field for many laboratories. Our ongoing studies revealed that ST32da treatment inhibited human primary pre-adipocyte differentiation and promoted browning (Supplementary Fig. 10). In the future, subcutaneous WAT harvested from human tissue biopsies may be first treated with ST32da and then expanded and induced to differentiate into brown adipocytes before their implantation as an autologous transplantation, thus enhancing the energy expenditure and improving glucose metabolism and insulin resistance in patients with obesity. In conclusion, ST32da, as an ATF3 inducer, could restore the positive balance of lipolysis/lipogenesis and increase the amount of functional BAT without central nervous system repression on dietary intake. ATF3 inducer could be a novel class of anti-obesity drug to treat diet-induced obesity and related metabolic disorders.

## Methods

### Animal studies

*ATF3*^–/–^ mice were kindly provided by Dr. Tsonwin Hai as described^58^. The *ATF3*^–/–^ allele was backcrossed into C57BL/6 mice for at least seven generations before the experiments. To assess metabolic parameters, 4-week-old male wild-type and *ATF3*^−/−^ mice were fed a chow diet (normal diet, ND, 8 % kcal from fat) or high-fat diet (HFD, 45% kcal from fat). Body weight, serum TG level, glucose tolerance, and insulin sensitivity were measured. To monitor the effects of i.p. or oral ST32da treatment, 6-week-old wild-type mice were fed a HFD with or without i.p. ST32da or oral ST32da for 12 and 16 weeks. Body weights and food intake were measured every week throughout the experiments. Metabolic parameters such as insulin sensitivity, glucose tolerance and liver weight were measured at the end of the treatment. Body composition was measured by using a TD-NMR analyzer (Minispec LF-50; Bruker Optics). All procedures were performed according to the protocols approved by the Institutional Animal Care and Utilization Committee, Academia Sinica, Taipei, Taiwan.

### Glucose tolerance test and insulin tolerance test

Glucose tolerance test, mice were fasted overnight for 16 h before receiving an i.p. administration of 1.5 g glucose kg^−1^ body weight in saline. Plasma glucose levels were measured from the tail blood at 0, 15, 30, 45, 60, and 120 min after glucose injection. Mice underwent insulin tolerance test after 4 h of fasting. Mice were i.p. injected with 0.75 Unit insulin kg^−1^ body weight in saline. Plasma glucose levels were measured from the tail blood at 0, 15, 30, 45, and 60 min after insulin injection. Plasma glucose levels collected from tail veins were determined by using a commercially available glucose meter (OneTouch Ultra blood glucose meter, LifeScan, Milpitas, CA).

### Cold exposure

For testing resistance to cold exposure, mice were individually caged and exposed to 4 °C with free access to water. Core body temperature was monitored by using a rectal thermometer (KN-91, NATSUME) at the beginning and every 2 h after the start of cold exposure. Images were taken after cold exposure with use of an infrared thermographic camera (F30s, NEC Avio Infrared Technologies, Tokyo)

### Indirect calorimetry measurements

Mice were measured after 9 weeks on a HFD by using an 8chamber LabMaster Calorimetry Module (TSE‐Systems GmbH) with one mouse per chamber. After acclimatization for 72 h, the O_2_ consumption (VO_2_, ml h^−1^ kg^−1^), CO_2_ production (VCO_2_, ml h^−1^ kg^−1^), respiratory quotient (ratio of VCO_2_/VO_2_) and energy expenditure were determined. VO_2_ and VCO_2_ were recorded every 1 for 48 h. Energy expenditure was calculated as the product of the calorific value of oxygen (3.815 + 1.232 × respiratory quotient) and the volume of O_2_ consumed.

### Analysis of serum parameters

The levels of serum BUN, creatinine, glucose, TG, GOT, and GPT were measured by sing Spotchem EZ SP 4430 (ARKRAY, Kyoto, Japan).

### Adipokine arrays

The serum levels of adipokines were analyzed by using Proteome Profiler Mouse Adipokine Array Kit (R&D Systems, Minneapolis, MN, USA). Blots were developed by using an enhanced chemiluminescence and a Fuji Film Imaging System (Application Note LAS-4000; Fuji film, Tokyo). Densitometry of blots involved use of the Gel-Pro Analyzer software.

### Histology, adipocyte size and adipocyte number

For hematoxylin and eosin (H&E) staining, dissected tissues of kidney, heart and adipose tissue were fixed in 4% paraformaldehyde overnight at 4 °C and paraffin-fixed before sectioning and staining. The stained sections of WAT were analyzed by using Image J. For adipocyte size measurements, 20 consecutive fat cells of the gonadal fat pad from mice were selected for measurement of area. The adipocyte number of the gonadal fat pad was calculated as the fat pad volume divided by the average fat cell volume.

### Production of recombinant AAV carrying *ATF3*

This procedure was described previously^24^. Full-length *ATF3* was obtained by PCR amplification from a human complementary cDNA library and cloned into XbaI/HindIII sites of the pAAV-MCS vector. A three-plasmid cotransfection method was used to produce the AAV virus^59^. The plasmids used in transfection included the AAV-CMV-*ATF3* plasmid with the gene driven by the CMV promoter, which carried the promoter-driven transgene flanked by AAV inverted terminal repeats; the helper plasmid, which contained helper genes from the adenovirus; and the pseudotyped AAV packaging plasmid containing the AAV8 serotype capsid gene coupled with the AAV2 rep gene. The AAV8-GFP (control) or AAV8-*ATF3* (experimental group) was purified twice by caesium chloride gradient ultracentrifugation, and the titers of vector genome particles were determined as described^60^. The recombinant viruses with 1 × 10^12^ viral particles in 30 µl phosphate buffered saline (PBS) were injected into a mouse-tail vein after 5 and 7 weeks of HFD feeding.

### Plasmid constructs

The *ATF3* sequence was subcloned into pcDNA3.1. The −3.6 kb *ATF3*-luc was generated by PCR amplification of the target region from genomic DNA and inserting into KpnI/NheI sites of the pGL4.17 vector (Promega). The 2.9-, 2.7-, 2.5-, 2-, and 1-kb *ChREBP*-luc promoters were generated by PCR amplification of the target region from genomic DNA and inserting into KpnI/EcoRV sites of the pGL4.17 vector (Promega). The 2.9-, 2- and 1-kb *FABP4*-luc promoters were generated by PCR amplification of the target region from genomic DNA and inserting into KpnI/NheI sites of the pGL4.17 vector (Promega). The SCD1 expression vector was from GeneCopoeia.

### Cell culture

3T3-L1 cells and human pre-adipocyte cells were used in this study. 3T3-L1 cells were maintained in Dulbecco’s modified Eagle’s medium supplemented with 10% calf serum, 100 U ml^−1^ penicillin, and 0.1 mg ml^−1^ streptomycin. Human pre-adipocyte cells were maintained in pre-adipocyte growth medium. For transient transfection assays, cells were seeded at 1.15 × 10^4^ cells cm^−2^. After 24 h, cells were transiently transfected with pcDNA-*ATF3* and shRNA-*ATF3* by using Maestrofectin transfection reagent (Omics Bio). After 48-h incubation, transfection efficiency was determined by real-time PCR and western blot analysis. For 3T3-L1 differentiation experiments, 2 days after 3T3-L1 cells reached confluence (referred to as day 0), 3T3-L1 cells were induced to differentiate in culture medium supplemented with 5 μg ml^−1^ insulin, 0.5 mM 3-isobutyl-1-methylxanthine, 1 μM dexamethasone for 2 days and then maintained in culture medium supplemented with 5 μg ml^−1^ insulin. For human pre-adipocyte differentiation experiments, 2 days after the human pre-adipocyte cells reached confluence (referred to as day 0), human pre-adipocytes were induced to differentiate in adipocyte differentiation medium for 14 days. For analyzing the effect of ST32da on cell differentiation, 3T3-L1 cells were treated with ST32da for 8 days of differentiation and human pre-adipocytes were treated with ST32da 14 days of differentiation.

### Oil-red O staining

After 8 and 14 days of pre-adipocyte differentiation in 3T3-L1 cells and human preadipocytes, respectively, differentiated adipocytes were washed twice with PBS and fixed for 1 h in 10% formalin. Cells were then stained with Oil-red O working solution for 1 h. Cells were washed four times with distilled water before microscopy analysis. Stained Oil-red O was eluted with 100% isopropanol (v/v) and quantified by measuring the optical absorbance at 500 nm.

### Western blot analysis

Cells were washed in cold PBS and lysed in RIPA buffer on ice. Adipose tissues (100 mg) were homogenized by using a tissue ruptor (Qiagen). Cell lysates and tissue homogenates were centrifuged at 14,000 rpm for 15 min at 4 °C and the infranatant was collected. Protein concentrations were determined by Bradford method. Proteins were resolved by SDS-PAGE and transferred to a PVDF membrane (Merck Millipore). The following antibodies were used: anti-ATF3 (cat# sc-188; 1:500; Santa Cruz Biotechnology), anti-UCP1 (cat# ab10983; 1:1000; Abcam), anti-ChREBP (cat# 81958; 1:1000; Abcam), anti-SCD1 (cat# 19862; 1:1000; Abcam), anti-adiponectin (cat# ab22554; 1:1000; Abcam), anti-actin (cat# sc-47778; Santa Cruz Biotechnology), anti-GAPDH (cat# ab8245; 1:10000; Abcam).

### Real-time PCR

Total RNA was extracted from cultured cells or adipose tissues by using Trizol reagent (Invitrogen) and RNA was reverse transcribed to cDNA with the iScript cDNA Synthesis Kit (Biorad). Real-time quantitative PCR analysis involved using the ABI PRISM 7700 Sequence Detection System (Applied Biosystems, Grand Island, NY) with SYBR green (Biorad). Sequences of primers used for real-time PCR are in Supplementary Tables, 2 and 3.

### In vitro promoter assay

For in vitro promoter assay, 3T3-L1 cells were transfected with or without pGL4.17-*ChREBP* (or pGL4.17-*FABP4*) and pcDNA-*ATF3* for 24 h before harvesting for luciferase assay. Firefly luciferase activity was determined and normalized to Renilla luciferase activity. Data shown represent mean and SEM from three independent experiments.

### Stable transfection

pcDNA-*ATF3* and pGL4.17-*ATF3* vectors were stably transfected into 3T3-L1 pre-adipocytes by using Maestrofectin transfection reagent (Omics Bio). Transfected cells were selected in 1000 µg ml^−1^ G418 for 4 weeks, then several stable clones were established. pcDNA-*ATF3* stable clones were confirmed by real-time quantitative PCR and western blot analysis. The pGL4.17-*ATF3* stable clones were confirmed by luciferase activity and compared to pGL4.17 control stable clones. Furthermore, pGL4.17-*ATF3* stable clones were used for further drug screening.

### Chromatin immunoprecipitation assay

3T3-L1 cells were fixed in 1% formaldehyde and chromatin immunoprecipitation (ChIP) was performed according to the Upstate protocol (Millipore). Chromatin was immunoprecipitated with anti-ATF3 antibody (Santa Cruz Biotechnology). The purified DNA was detected by standard PCR. Primers are in Supplementary Table 4.

### Measure serum levels of adiponectin, ICAM1, and resistin

Serum levels of adiponectin, ICAM1 and resistin were measured using an ELISA (Abcam, cat# ab226900 for mouse adiponectin ELISA kit; cat# ab252355 for mouse ICAM1 ELISA kit; cat# ab205574 for mouse resistin ELISA kit) according to the manufacturer’s instructions.

### Microarray data sources

We use microarray experiment data obtained from GEO (Gene Expression Omnibus, https://www.ncbi.nlm.nih.gov/geo/) at NCBI, including nonalcoholic fatty liver disease (accession no. GDS4881, liver from obese individuals) and morbidly obese subjects (accession no. GDS3679, adipose tissue from obese individuals; accession no. GDS368, muscle tissue from obese individuals; accession no. GSE69039, monocytes from obese individuals).

### Statistical analyses

Values are expressed as means ± SEM from at least three experiments. The statistical significance was analyzed usingANOVA followed by the Tukey test for the in vivo experiments. Samples from the patients were analyzed using the rank sum test. A value of *p* < 0.05 was considered statistically significant.

### Reporting summary

Further information on research design is available in the Nature Research Reporting Summary linked to this article.

## Supplementary information

Supplementary Information

Description of additional supplementary files

supplementary data 1

supplementary data 2

supplementary data 3

Reporting Summary

## Data Availability

All data generated or analyzed during this study are included in this published article. Full blots are shown in Supplementary Information. The source data underlying plots presented in main figures are shown in Supplementary Data 1.
