# Supplementary material for: Adipocyte browning and resistance to obesity in mice is induced by expression of ATF3
Source: Commun Biol. 2019 Oct 24;2:389. doi: 10.1038/s42003-019-0624-y (PMC6813364; doi:10.1038/s42003-019-0624-y)
Supplement: Supplementary file 1 — Supplementary Information [file 42003_2019_624_MOESM1_ESM.pdf]

Supplementary Fig. 1 Additional examples of representative images shown in the main and supplementary figures. **a** Representative H&E staining of inguinal WAT (iWAT), epididymal WAT (eWAT) and BAT in wild-type and *ATF3*$^{-/-}$ after HFD-induced obesity. **b**. Oil-red O staining of liver in wild-type and *ATF3*$^{-/-}$ after HFD-induced obesity . Scale bar = 100 μm. **c** ATF3 protein level in iWAT and BAT of wild-type and *ATF3*$^{-/-}$ mice after HFD feeding for 12 weeks. **d** Representative immunofluorescence images of adiponectin (red IF) and ICAM-1 (green IF) in wild-type and *ATF3*$^{-/-}$ mice. Yellow scale bar indicated the size of adipocyte tissues **e** Serum protein levels of adipokine and inflammation-related genes in wild-type and *ATF3*$^{-/-}$ mice after HFD feeding for 8 weeks by adipokine assays; Gel-Pro Analyzer software was used for densitometry of blots. **f** ATF3 protein level in different organs in WT and *ATF3*$^{-/-}$ mice with or without restoration of ATF3 expression by adeno-associated virus containing *ATF3* and GFP as control. **g, h** Protein levels of ChREBP, SCD1, UCP1 and adiponectin in iWAT or in BAT (n = 3 per group) after *ATF3*$^{-/-}$ and wild-type mice 12 weeks HFD feeding. **i** Protein levels of ChREBP and SCD1 in WT and *ATF3*$^{-/-}$ mice with or without restoration of ATF3 expression by adeno-associated virus containing *ATF3* and GFP as control. **j** H&E staining of inguinal WAT, epididymal WAT, and BAT fat depots (n = 3 per group) in HFD-induced obesity mice following oral administration of ATF3 inducer, ST32da.

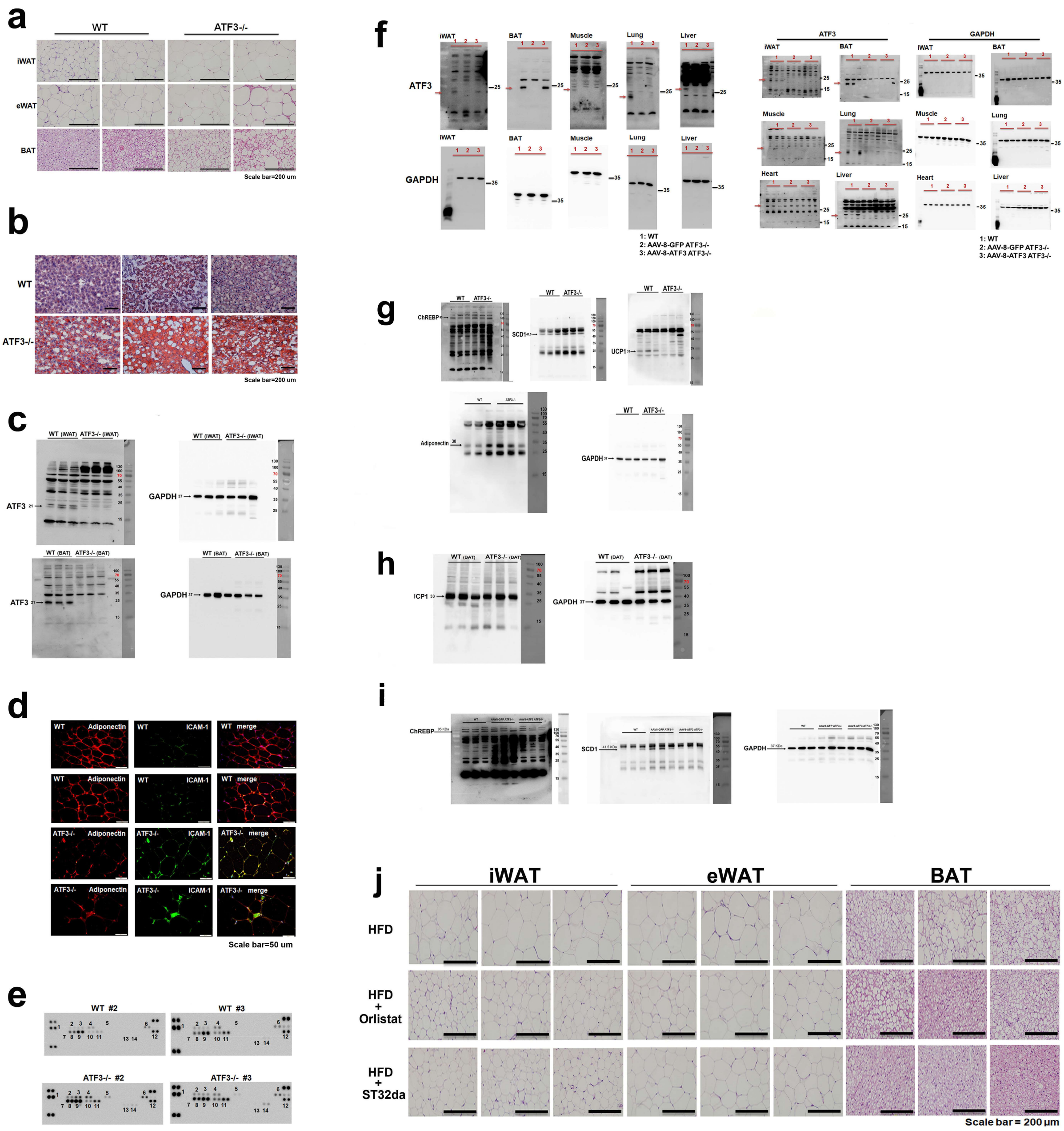
a
WT
ATF3-/-
iWAT
eWAT
BAT
Scale bar=200 um
b
WT
ATF3-/-
Scale bar=200 um
c
WT (iWAT)
ATF3-/- (iWAT)
ATF3
GAPDH
WT (BAT)
ATF3-/- (BAT)
d
WT
Adiponectin
ICAM-1
merge
ATF3-/-
Scale bar=50 um
e
WT #2
WT #3
ATF3-/- #2
ATF3-/- #3
f
iWAT
BAT
Muscle
Lung
Liver
Heart
ATF3
GAPDH
1: WT
2: AAV-8-GFP ATF3-/-
3: AAV-8-ATF3 ATF3-/-
g
ChREBP
SCD1
UCP1
Adiponectin
h
ICP1
i
j
iWAT
eWAT
BAT
HFD
HFD + Orlistat
HFD + ST32da
Scale bar = 200 µm

Supplementary Fig. 2 ATF3 protein level in different organs in WT and *ATF3*$^{-/-}$ mice with or without restoration of *ATF3* expression by adeno-associated virus containing *ATF3* and GFP as control. $n = 3$ per group

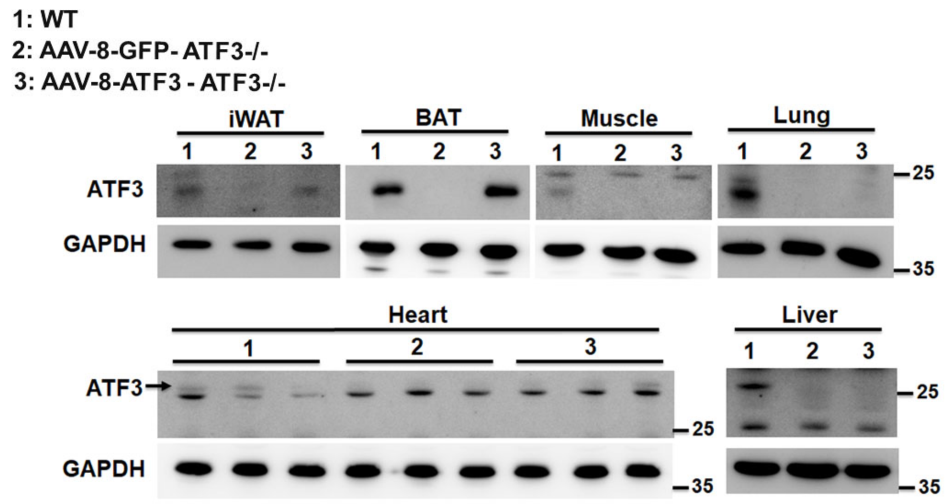

Supplementary Fig. 3 Protein levels of ChREBP and SCD1 in WT and *ATF3*$^{-/-}$ mice with or without restoration of *ATF3* expression by adeno-associated virus containing *ATF3* and GFP as control. $n = 3$ per group

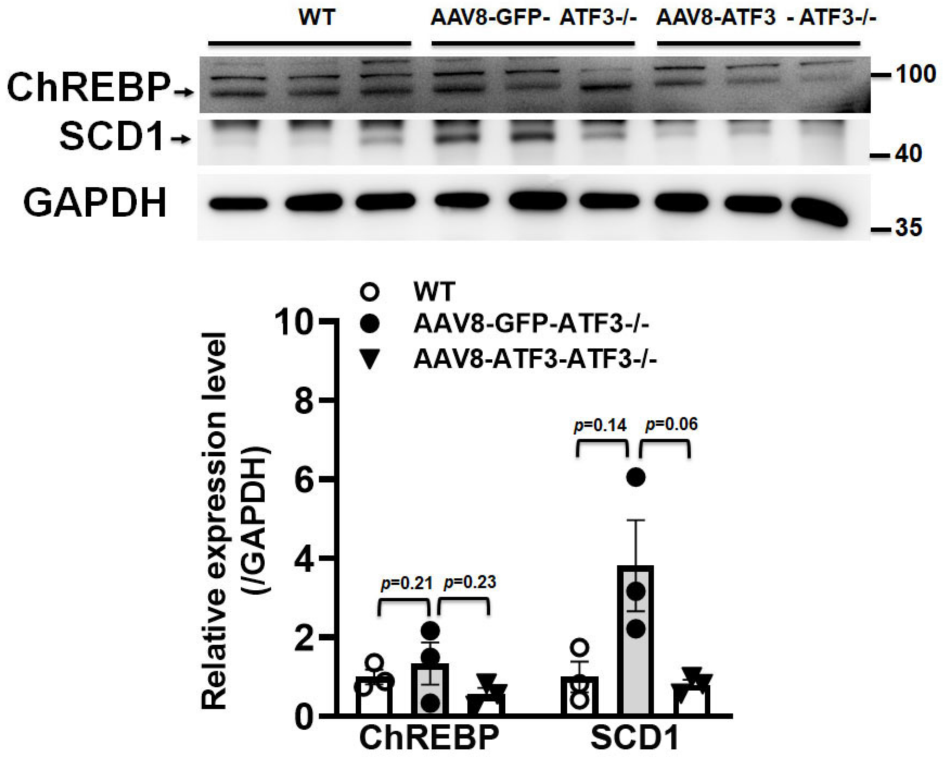

Supplementary Fig. 4 Positive correlations between ATF3 and HSL and CIDEA were validated in adipose tissue of patients by using Gene Expression Omnibus (GEO) and the GDS3679 dataset. **a** Correlation between ATF3 and HSL expression (Pearson correlation coefficient *r* =0.199, *p* =0.0329); **b** Correlation between ATF3 and CIDEA expression (Pearson correlation coefficient *r* =0.1938, *p* =0.0403) in human adipose tissue. For **a,** *n* = 23 in HSL and ATF3. For **b,** *n* = 31 in CIDEA and ATF3.

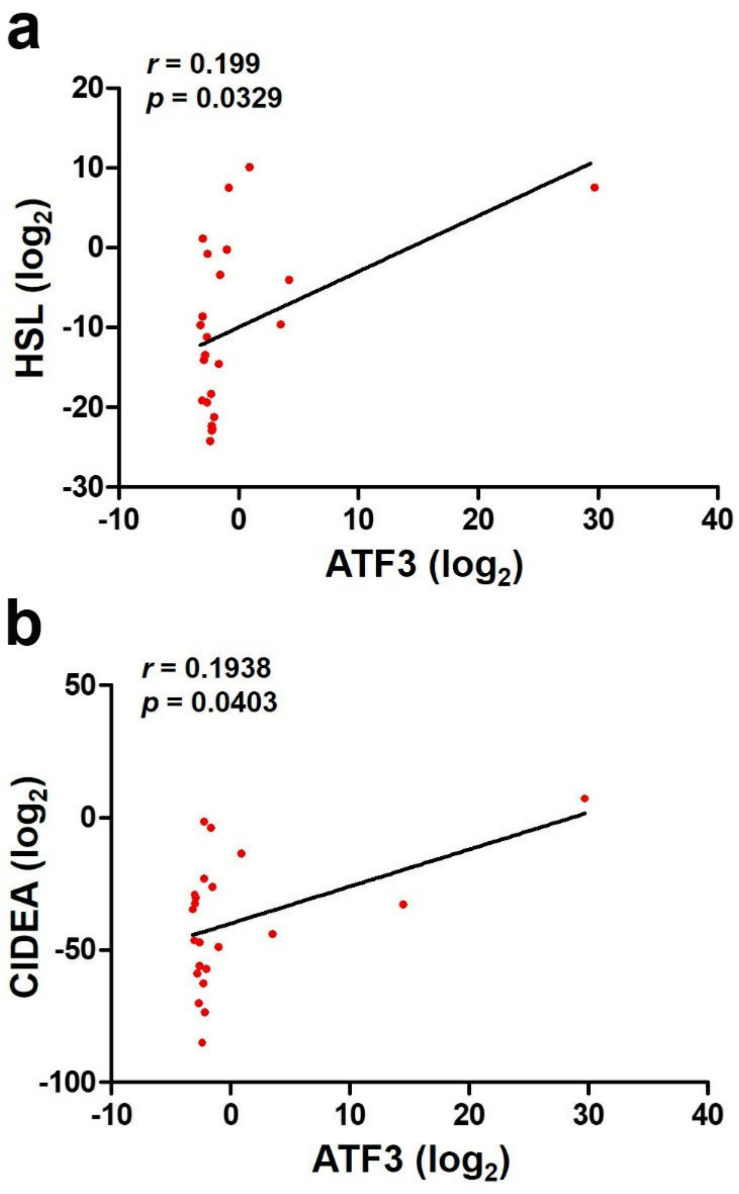

Supplementary Fig. 5 Overexpression of ATF3 decreased oil droplet deposition in 3T3-Ll cells after 8 days of differentiation. **a** Protein level of ATF3 in 3T3-L1 cells transfected separately with pcDNA-*ATF3*, shRNA-*ATF3* or control vector. **b** Oil-red O staining of control and ATF3 overexpressing 3T3-L1 adipocytes differentiated for 8 days. The light-phase microscopy images of differentiated cells (scale bar = 200 μm) and relative quantification of adipocyte differentiation. For **a**, **b**, n = 3 per group. Data are presented as mean ± SEM and *$p < 0.05$ compared to pcDNA.

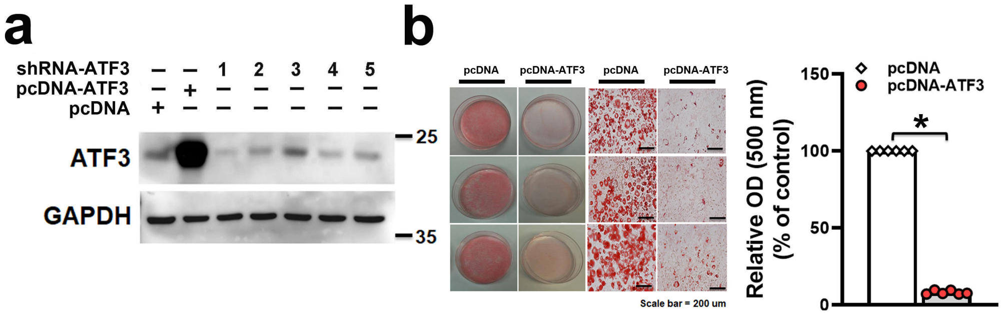

Supplementary Fig. 6 Characteristics of the investigated compound, ST32da. The structure of ST32da was determined by UFLC-MS/MS system.

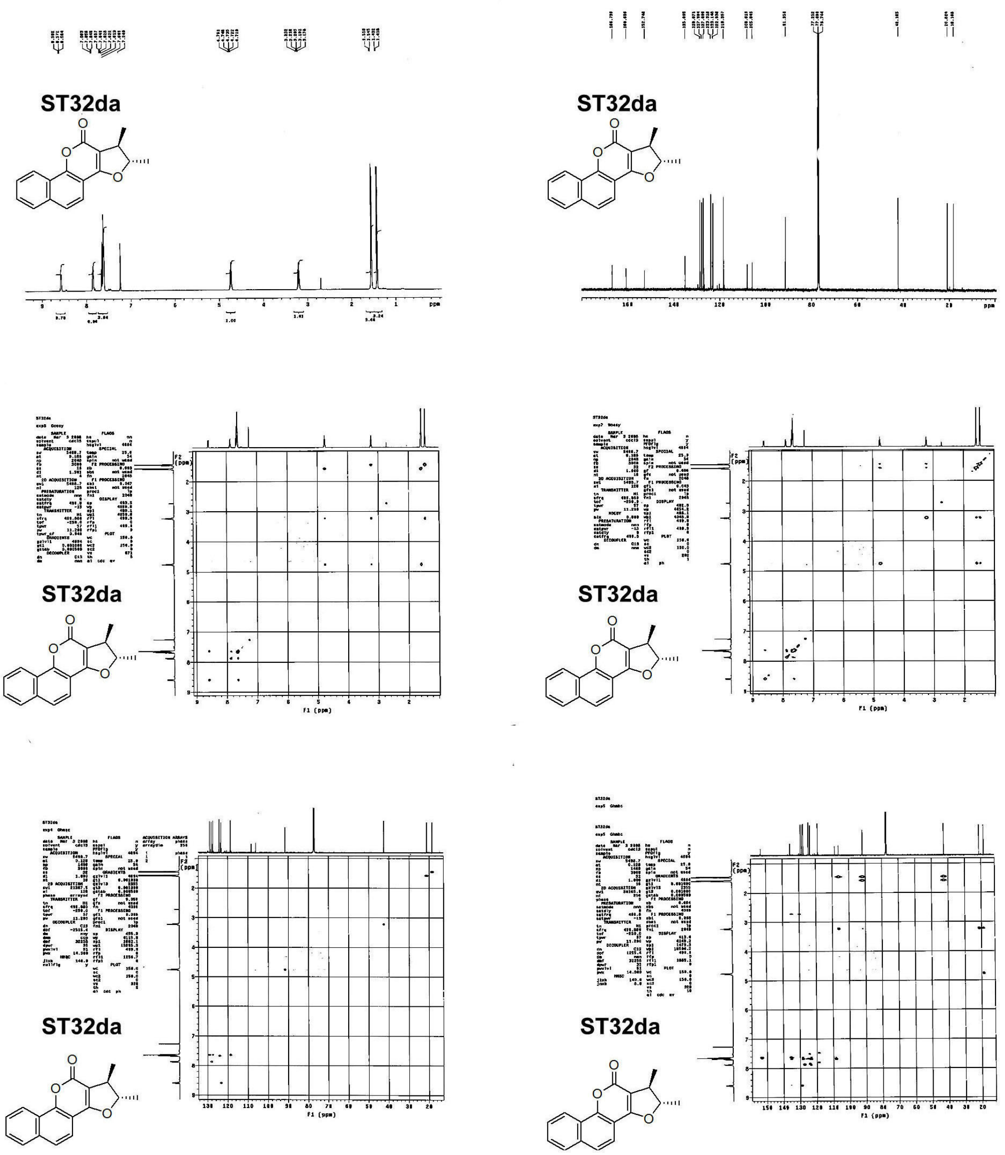

Supplementary Fig. 7 Physiological effects of ATF3 inducer, ST32da, on mouse heart, kidney and liver. Analysis of WT mice fed with HFD for 12 weeks with or without i.p. ST32da treatment at a dose of 1 mg kg^{-1} per day or 2 mg kg^{-1} per day. **a** Serum levels of BUN, creatinine, glucose and triglyceride (TG). **b** Serum levels of GOT and GPT. **c** Liver weight. **d** H&E staining of heart and kidney histology. For **a**, HFD (n = 7), HFD+1 mg kg^{-1} per day (n = 7), HFD+2 mg kg^{-1} per day (n = 4). For **b**, n = 6 per group. For **c**, HFD (n = 9), HFD+1 mg kg^{-1} per day (n = 7), HFD+2 mg kg^{-1} per day (n = 6). For **d**, n = 3 per group. Scale bar for image **d**: 100 µm. Data are presented as mean ± SEM and *p < 0.05 compared to HFD group.

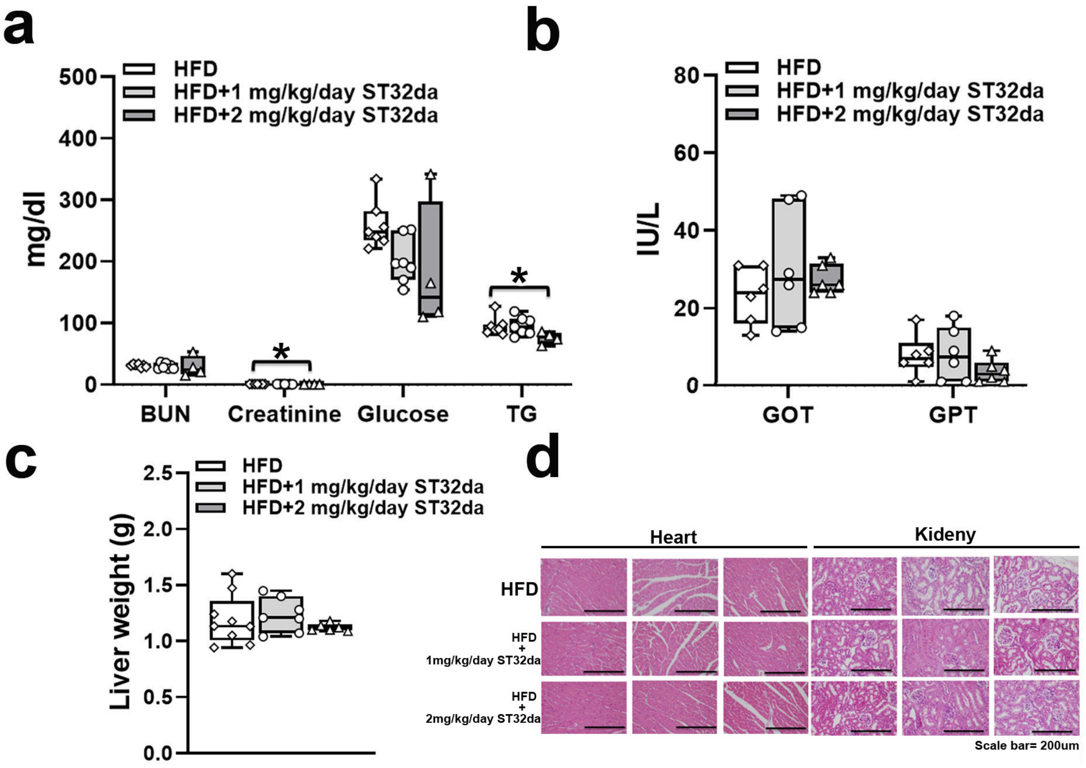

Supplementary Fig. 8 The effect of ATF3 inducer, ST32da, in HFD-induced obese *ATF3*$^{-/-}$ mice. Analysis of *ATF3*$^{-/-}$ mice fed a HFD for 12 weeks with or without ATF3 inducer, ST32da, at 2 mg kg-1 per day. **a** Body weight. **b** Food intake. **c** Glucose tolerance test (GTT). **d** Insulin tolerance test (ITT). **e** Change in adipose tissue depot weight in BAT and WAT. **f** mRNA level of ChREBP and SCD1 in iWAT. **g** Liver weight. **h** H&E staining of iWAT and BAT; adipocyte size (um^2) and number per area (mm^2). Scale bar = 100 μm. For **a**, *ATF3*$^{-/-}$ (n = 5), *ATF3*$^{-/-}$ +2 mg kg^{-1} per day ST32da (n = 4). For **b**, n = 12 per group. For **c**, **d**, *ATF3*$^{-/-}$ (n = 5), *ATF3*$^{-/-}$ +2 mg kg^{-1} per day ST32da (n = 4). For **e**, *ATF3*$^{-/-}$ (n = 6), *ATF3*$^{-/-}$ +2 mg kg^{-1} per day ST32da (n = 5). For **f**, n = 5 per group. For **g**, *ATF3*$^{-/-}$ (n = 6), *ATF3*$^{-/-}$ +2 mg kg^{-1} per day ST32da (n = 5). For **h**, n = 3 per group in H& E staining; n = 4 per group in adipocyte size; *ATF3*$^{-/-}$ (n = 4), *ATF3*$^{-/-}$ +2 mg kg^{-1} per day ST32da (n = 3) in adipocyte number/area (mm^2). Data are presented as mean ± SEM.

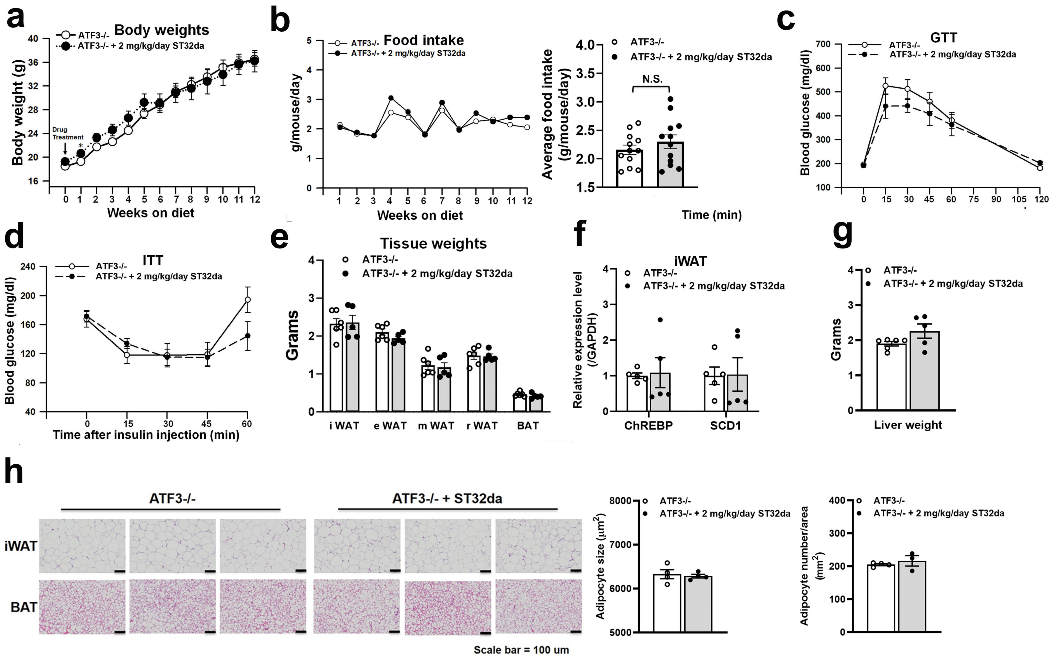

Supplementary Fig. 9 The effect of ATF3 inducer, ST32da, on serum adiponectin levels in HFD-fed *ATF3*$^{-/-}$ mice. *n* = 3 per group. Data are presented as mean ± SEM.

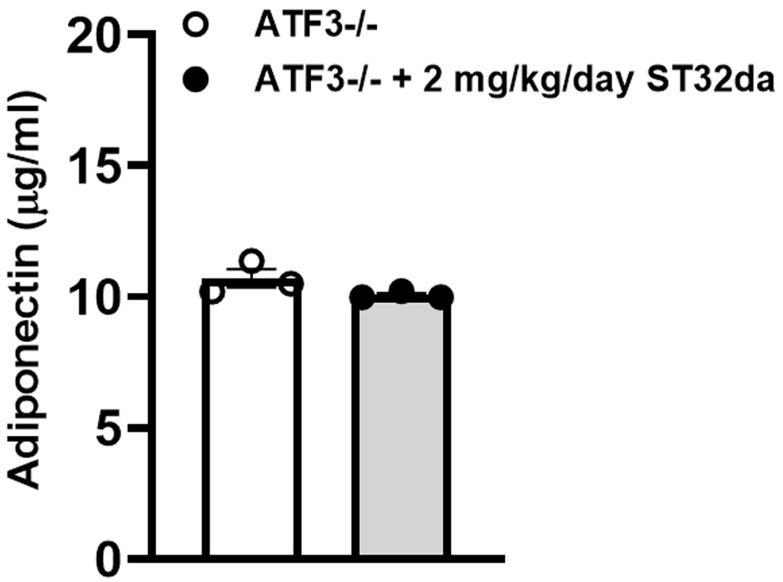

Supplementary Fig. 10 ATF3 inducer, ST32da, inhibited adipogenesis and induced browning in human primary adipocytes. **a** Photograph of Oil Red O staining in differentiated human primary adipocytes in the absence or presence of ATF3 inducer ST32da (50 and 100 uM) for 14 days. **b**, **c** Gene expression levels of adipogenic, lipogenic, lipolytic, brown (BAT) and β-oxidation (β-oxi) genes were measured by real time PCR after 7 days (**b**) and 14 days (**c**) of ST32da treatment during human preadipocyte differentiation, normalized to GAPDH and relative to control. For **a**, *n* = 3 per group. For **b**, control (*n* = 6), 50 uM (*n* = 4), 100 uM (*n* = 4). For **c**, *n* = 5 per group. Data are presented as mean ± SEM and *$p < 0.05$ compared to control group.

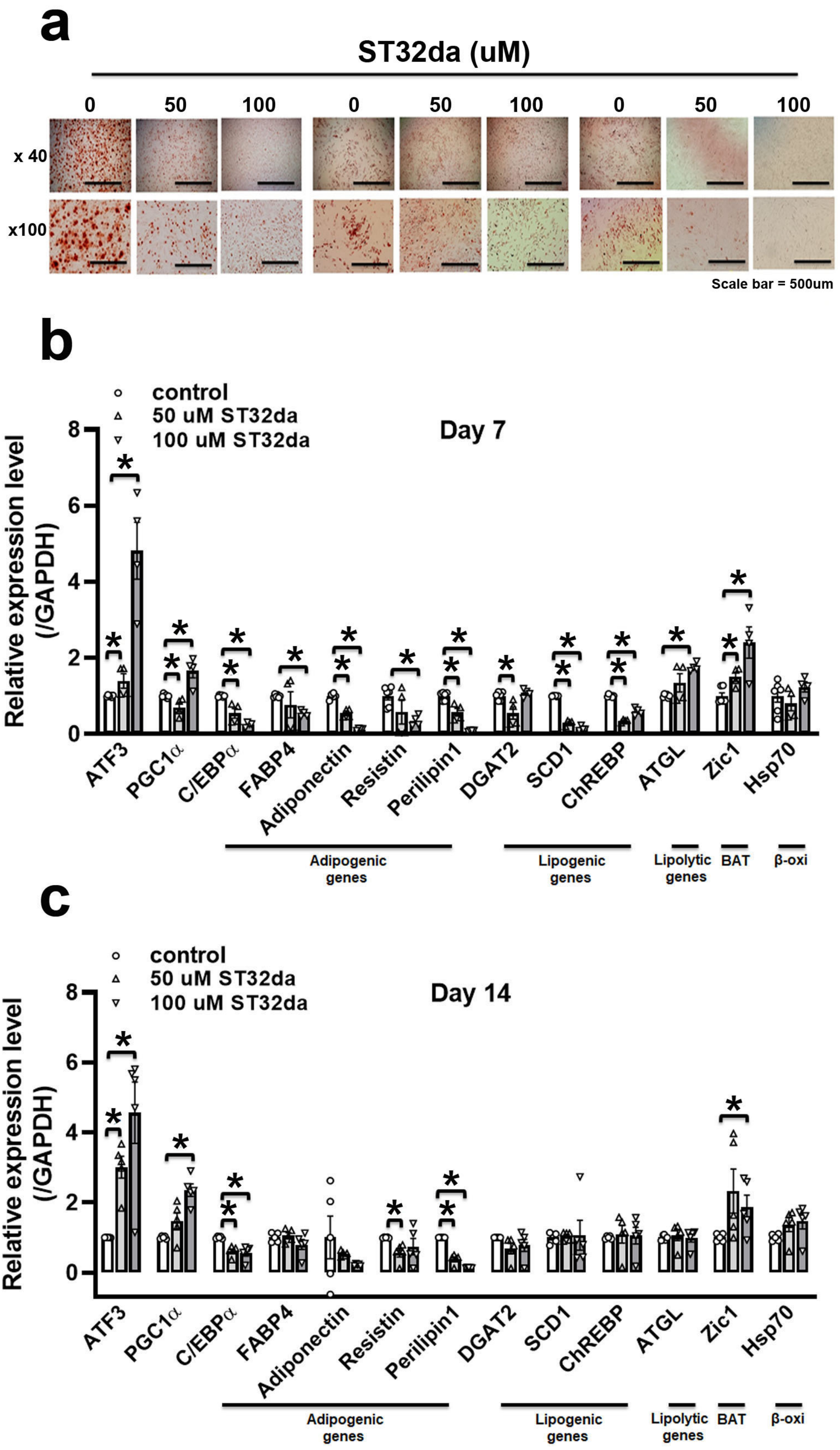
a
ST32da (uM)
0
50
100
0
50
100
0
50
100
x 40
x100
Scale bar = 500um
b
control
50 uM ST32da
100 uM ST32da
Day 7
Relative expression level (/GAPDH)
0
2
4
6
8
ATF3
PGC1α
C/EBPα
FABP4
Adiponectin
Resistin
Perilipin1
DGAT2
SCD1
ChREBP
ATGL
Zic1
Hsp70
Adipogenic genes
Lipogenic genes
Lipolytic genes
BAT
β-oxi
c
control
50 uM ST32da
100 uM ST32da
Day 14
Relative expression level (/GAPDH)
0
2
4
6
8
ATF3
PGC1α
C/EBPα
FABP4
Adiponectin
Resistin
Perilipin1
DGAT2
SCD1
ChREBP
ATGL
Zic1
Hsp70
Adipogenic genes
Lipogenic genes
Lipolytic genes
BAT
β-oxi

Supplementary Table 1. Possible ATF3 inducers, obtained from herbal compound library, screened by ATF3 platform.

Compounds	(Exp./Con.)	Compounds	(Exp./Con.)	Compounds	(Exp./Con.)
tBHQ (positive control)	1.929658424	TYC05	0.354005	TYC517	0.360332
ST32ba	1.98956385	TYC234	0.286002	TYC519	0.488055
ST32c	2.497351472	TYC226	0.448314	TYC702	0.438284
ST32da	2.181297514	TYC229	0.31815	CC-A	0.346534
ST32db	2.175265937	TYC236	0.285824	CC-B	0.373831
ST64	1.963102234	TYC239	0.345438	CC-E	0.362694
WTS16	1.547130489	TYC238	0.48942	CC-F	0.40428
WTS08	2.023839359	TYC240	0.395818	CH-206-G	0.418966
WTS09	1.740977273	TYC241	0.31165	CYZ02	0.461929
ST74	1.814256378	CCU07	0.423129	CYZ03	0.32093
SM102	1.877530021	TYC302	0.292307	CYZ05	0.447353
SM104	1.678314535	TYC321	0.400383	CYZ07	0.323651
Sa-22	2.004158739	TYC251	0.447515	CYZ09	0.402041
Sa-26	2.095282268	TYC307	0.29844	CYZ11	0.387415
Sa-32	1.547084467	TYC305	0.359376	CYZ12	0.392376
Sa-33	1.963227779	TYC323	0.478463	CYZ14	0.379787
L8-4-7-1-2	2.067197683	CH-183-4-C	0.262024	CYZ17	0.438328
F3-4-175-1	2.040957351	Syd56	0.422594	TYC263	0.461696
CH-183-4-B	2.353515322	Syd58	0.396068	107-206B	0.320762
WLT-01090	2.381909031	TYC506	0.410256	CM-Hex-2-5-4-4	0.22683
		TYC509	0.439928	CM-Hex-2-5-4-5	0.333034
		TYC516	0.33713	WLT-01021	0.36311

Supplementary Table 2. Primers 1.

Gene name	Forward Primer 5'-3'	Reverse Primer 5'-3'
mATF3	CTCCTGGGTCACTGGTATTTG	CCGATGGCAGAGGTGTTTAT
mC/EBPα	GTAACCTTGTGCCTTGGATACT	GGAAGCAGGAATCCTCCAAATA
mC/EBPβ	CTTGATGCAATCCGGATCAAAC	CCCGCAGGAACATCTTTAAGT
mPPARγ1	GGACTGTGTGACAGACAAGATTTG	CTGAATATCAGTGGTTCACCG
mPPARγ2	CTGGCCTCCCTGATGAATAAAG	AGGCTCCATAAAGTCACCAAAG
mFABP4	GCTCCTCCTCGAAGGTTTAC	CCCACTCCCACTTCTTTCAT
mAdiponectin	AAGGGCTCAGGATGCTACTGTT	AGTAACGTCATCTTCGGCATGA
mLeptin	GGTTGATCTCACAATGCGTTTC	TGGGAGACAGGGTTCTACTT
mResistin	CTAAGCTGAGGGTCTGGAAATG	CACACACCCTTCTCCACTAAAG
mPerilipin1	AGGCTGTCTCCTCTACCAAA	CCACAGTGTCTACCACGTTATC
mPerilipin2	CGTCTGTCTGGACCGAATAAAG	CACACGCCTTGAGAGAAACA
mACC1	TGATGGTGGCCTGCTCTTGTCTTA	CAGCAAACACATGTCCGCCATCTT
mACC2	ACCCACTGTCTTCCAATGACACCT	TCAGCTGTCTCTTGATGTGTGCCT
mFAS	AGACCCGAACTCCAAGTTATTC	GCAGCTCCTTGTATACTTCTCC
mDGAT1	GGCCTTACTGGTTGAGTCTATC	GTTGACATCCCGGTAGGAATAA
mDGAT2	GAAGGGCTTCTCTTCTCTTCAC	CTTTCTCCCAACGCCTCATAA
mSCD1	TGGGTTGGCTGCTTGTG	GCGTGGGCAGGATGAAG
mChREBP	TGTTCAGCATCCTCATCCGACCTT	TGAGTTGGCGAAGGGAATTCAGGA
mSREBP1	GCGCCATGGACGAGCTG	TTGGCACCTGGGCTGCT
mATGL	CATCCGTGGCTGTCTACTAAAG	GACGTTCTCTCCGTCTGAAAC
mHSL	GGACGGTCCTAGGTTTGAATAC	GATGGGAAGGTCTGTGGTTAC
mMGL	GACAGAAAGAGTGTGGGAAGAG	CTGAGCACAGTAGTCTGGAATG
mPGC1α	CTAGCCATGGATGGCCTATTT	GTCTCGACACGGAGAGTTAAAG
mUCP1	GAGGTCGTGAAGGTCAGAATG	AAGCTTTCTGTGGTGGCTATAA
mPrdm16	CAGCCATACAGGTGCAAGTA	GAACGGCTTCTCTTTGTTGTG
mDio2	CTTCCTCCTAGATGCCTACAAAC	TCTCCGAGGCATAATTGTTACC
mZic1	ATATGCGCCAACCCATCAA	TCGTGCATGGTGCTGAAA
mCIDEA	GCAACCAAAGAAATCGGGAATAG	CTCGTACATCGTGGCTTTGA
mElovl3	TACATCTGGAGGCAGGAGAA	GGTGGAAGAAGTGAGCGAATAG
mCD137	CTGGCCCTGATCTTCATTACTC	CTGCTCCAGTGGTCTTCTTAAA
mTbx1	CCCGATTCCATGTTGTCTATGT	GTGAAGCGTGTCTCCTCAA
mCytC	GAGGATACCCTGATGGAGTATTTG	GCTATTAGGTCTGCCCTTTCTC
mCox4-1	AGTTGTACCGCATCCAGTTT	GCAGTGAAGCCAATGAAGAAC
mCox4-2	ACGAATGGAAGACAGTGATGG	GTCAGGGTGACAACCTTCTTAG
mMcad	GAGAAGAAGGGTGACGAGTATG	GGCTTTACTAGCGGGTACTTTA
mCpt1α	GAAGTGTCGGCAGACCTATTT	GTCCTCCTCTCTATATCCCTGTT

Supplementary Table 3. Primers 2.

mHSP70	TGGTGCTGACGAAGATGAAG	CGCTGAGAGTCGTTGAAGTAG
miNOS	GGAATCTTGGAGCGAGTTGT	CCTCTTGTCTTTGACCCAGTAG
mIL-6	CTTCCATCCAGTTGCCTTCT	CTCCGACTTGTGAAGTGGTATAG
mTNFα	TCTCATGCACCACCATCAAGGACT	ACCACTCTCCCTTTGCAGAACTCA
mGAPDH	GGAGCCAAACGGGTCATCATCTC	GAGGGGCCATCCACAGTCTTCT
hATF3	CTGGAAAGTGTGAATGCTGAAC	ATTCTGAGCCCGGACAATAC
hC/EBPα	GAAGTCGGTGGACAAGAACA	TCATTGTCACTGGTCAGCTC
hC/EBPβ	CGCGACAAGGCCAAGAT	GCTGCTCCACCTTCTTCTG
hPPARγ1	GCCTGCATCTCCACCTTATTA	ATCTCCACAGACACGACATTC
hPPARγ2	GCCTGCATCTCCACCTTATTA	ATCTCCACAGACACGACATTC
hFABP4	GGAAAGTCAAGAGCACCATAAC	GCATTCCACCACCAGTTTATC
hAdiponectin	CATTCCTGGGCTGTACTACTTT	GAGCATAGCCTTGTCCTTCTT
hLeptin	CCTTATCCAAGATGGACCAGAC	GTTCTCCAGGTCGTTGGATATT
hResistin	AAAGCTCTCTGTCTCCTCCT	ACCTCCTGGATCCTCTCATT
hPerilipin1	CCAGAGACACTGCGGAATTT	GTACTCCACCACCTTCTCAATG
hDGAT2	CTGGAGAACCTCATCAAGTATGG	CAAAGACATTGGCCGCAATAA
hSCD1	ACAACTACCACCACTCCTTTC	GGAGACTTTCTTCCGGTCATAG
hChREBP	GGAAGAATTTCAAAGGCCTCAAG	CTCTTCCTCCGCTTCACATAC
hSREBP1	GAGCCATGGATTGCACTTTC	AGCATAGGGTGGGTCAAATAG
hATGL	AACACCAGCATCCAGTTCA	TATCCCTGCTTGCACATCTC
hHSL	GATGGAAGTGCTATCGTCTCTG	AGTCAGTGGCATCTCAAAGG
hMGL	CTCATTTCGCCTCTGGTTCT	GCACAAGGTTGAGCACTTTC
hPGC1α	TGAACTGAGGGACAGTGATTTC	CCCAAGGGTAGCTCAGTTTATC
hUCP1	TCTCCACCAGGACAGTACAA	CAGGATCCAAGTCGCAAGAA
hZic1	GAGCGACAAGCCCTATCTTT	GGATTCGTGGACCTTCATGT
hCIDEA	GTGAAGGCCACCATGTATGA	TGTGCCCAGATAGATGAGAAAC
hElovl3	AGCGGCCACTCATCTTTAT	GCAGGCACTTTGTTCTTGTATC
hMcad	TGCTGGTGCTGTTGGATTAG	TGGTGCTCTACAAGTAGCTTTC
hHSP70	GACCTGCCAATCGAGAATCA	CCGCTCCTTCTCCAGTTTATC
hGAPDH	GGTGTGAACCATGAGAAGTATGA	GAGTCCTTCCACGATACCAAAG

Supplementary Table 4. Primers for ChIP assay.

ChREBP promoter	Forward Primer 5'-3'	Reverse Primer 5'-3'
Primer 1	TTGCTTTGGTTTGGAGACAGGTTCTCA	TCCCAGCACTGTGGAGTGCAGAG
Primer 2	CACAGTGCTGGGATTGAAGGTGTGA	GCGGACCCTGTGCCTCTCTACAGT
Primer 3	TGAATCACCACATCTGGCCACTTT	TGTGAGGCCTCTCTTTCCCACTAG
