# Supplementary material for: Adipocyte browning and resistance to obesity in mice is induced by expression of ATF3
Source: Commun Biol. 2019 Oct 24;2:389. doi: 10.1038/s42003-019-0624-y (PMC6813364; doi:10.1038/s42003-019-0624-y)
Supplement: Supplementary file 2 — Description of additional supplementary files [file 42003_2019_624_MOESM2_ESM.pdf]

Table legends for Supplementary data 1 (Raw data for main figures):

Raw data for Fig. 1 (Analysis of ATF3 expression level among liver, adipose tissue, muscle and blood monocytes from lean, obese and morbidly obese patients by NCBI GEO DataSets). **a** Homo-Liver. **b** Homo-Adipose tissue. **c** Homo-Muscle. **d** Homo-Blood monocyte.

Raw data for Fig. 2 (Loss of ATF3 in mice aggravated high fat diet (HFD)-induced obesity and metabolic dysfunction). **a** Body weight under normal diet and high fat diet. **b** Body composition (% of body weight). **c** TG level. **d** GTT. **e** ITT. **f** WAT and BAT fat-pad weight per body weight. **h** Adipocyte diameter, size, number per area, and perigonadal fat pad weight per body weight Adipocyte diameter (um) Adipocyte size (um^2) Adipocyte number per area (mm^2) perigonadal fat pad weight per body weight (%).

Raw data for Fig. 3 (Loss of ATF3 aggravated the expression of inflammation-related genes in HFD-induced obese mice). **c** Serum protein levels of adipokine and inflammation-related gene. **d** Serum protein level of adiponectin, ICAM-1 and resistin by ELISA assay. **e** Quantified real-time PCR analysis of mRNA levels of iNOS, IL-6, and TNFα in liver.

Raw data for Fig. 4 (Adeno-associated virus 8 (AAV8)-mediated expression of ATF3 reversed metabolic dysfunction in $ATF3^{-/-}$ mice). **a** Body weight (g). **b** TG level. **c** GTT. **d** ITT. **f** Adipocyte diameter (μm), size (μm^2), number per area (mm^2), and perigonadal fat pad weight per body weight (g).

Raw data for Fig. 5 ($ATF3^{-/-}$ mice showed dysregulated WAT/BAT balance). Analysis of $ATF3^{-/-}$ and wild-type mice after 12 weeks of HFD feeding. **a** Weight of iWAT, eWAT, mWAT, rWAT and BAT (g). **b** Analysis of gene expression of adipogenic, lipogenic, and lipolytic genes in iWAT. **c** Analysis of gene expression of brown (BAT), beige (Bei), mitochondria (Mito), and β-oxidation (β-oxi) markers in iWAT. **d** Analysis of expression of brown/mitochondria/β-oxidation markers in BAT. **e** Protein levels of ChREBP, SCD1, UCP1 and adiponectin in iWAT. **f** Protein level of UCP1 in BAT.

Raw data for Fig. 6 ($ATF3^{-/-}$ mice showed impaired energy metabolism and thermoregulation). **a** Body temperature and rectal temperature. **b** Body weight. **c** VO_2 (ml per kg per hr). **d** RER (VCO_2/VO_2). **e** Heat (kcal per kg per hr).

Raw data for Fig. 7 (ATF3-overexpressing 3T3-L1 adipocytes showed suppression of lipogenesis/adipogenesis and activation of mitochondrial, brown or beige fat programs). **a** Real-time PCR analysis of mRNA levels of adipogenic, lipogenic, and lipolytic genes (Day 2). **b** Real-time PCR analysis of mRNA levels of adipogenic, lipogenic, and lipolytic genes (Day 8). **c** Real-time PCR analysis of mRNA levels of BAT, beige (Bei), mitochondria (Mito), and β-oxidation (β-oxi) (Day 2). **d** Real-time PCR analysis of mRNA levels of BAT, beige (Bei), mitochondria (Mito), and β-oxidation (β-oxi) (Day 8). **e**. The expression level of ChREBP in iWAT. **f** The expression level of FABP4 in iWAT. **g** FABP4 promoter activity measured with or without overexpression of ATF3 in 3T3-L1 pre-adipocytes. **h** Overexpression of ATF3 repressed the ChREBP promoter activity of the p (-2980)/Luc reporter but not other reporters in 3T3-L1 preadipocytes. **k** Real-time PCR analysis of gene levels of brown (BAT), mitochondrial (Mi), beige (Bei), and β-oxidation (β-oxi) genes in ATF3-overexpressing 3T3-L1 preadipocyte stable clone with or without SCD1 transfection.

Raw data for Fig. 8 (Identification of ATF3 inducers and their functional assays). **b** Luciferase activity of stable clones of 3T3-L1 pre-adipocytes expressing pGL4.17-ATF3. **c** Luciferase activity measured in 3T3-L1 pre-adipocytes transfected with pGL4.17-ChREBP (p (-2980)/Luc reporter), then treated with ST32da or ST32db or ST32c. **e** Real-time PCR analysis of mRNA levels of adipogenic, lipogenic, and lipolytic genes (Day 2 and Day 8). **f** BAT, beige (Bei), mitochondria (Mito) and β-oxidation (β-oxi) gene expression with 2 and 8 days of ST32da treatment during 3T3-L1 differentiation.

Raw data for Fig. 9 (ATF3 inducer, ST32da, protects against HFD-induced obesity and metabolic dysfunction by promoting browning in vivo). **a** Body weight and food intake. **b** Change in adipose tissue depot weight in BAT and WAT. **d** Glucose tolerance test. **e** Insulin tolerance test. **f** Real-time PCR analysis of mRNA levels of ATF3, c-Jun, PGC-1α and UCP1. **g** Real-time PCR analysis of mRNA levels of brown (BAT) and beige (Bei), mitochondria (Mito), and β-oxidation (β-oxi) genes in iWAT. **h** Real-time PCR analysis of mRNA levels of adipogenic, lipogenic, and lipolytic genes in iWAT. **i** brown fat programs in BAT.

Raw data for Fig. 10 (Oral administration of ATF3 inducer, ST32da, is effective in preventing HFD-induced obesity). **a** Body weights and Food intake. **b** Variation of adipose tissue depot weight in BAT and WAT. **d** Serum parameters. **e** Liver weight. **f** Liver function. **g** Real-time PCR analysis of mRNA levels of ATF3, c-Jun, PGC-1α and UCP1 in iWAT. **h** Real-time PCR analysis of mRNA levels of brown (BAT), beige (Bei),

mitochondria (Mito), and β-oxidation (β-oxi) genes in iWAT. **i** Real-time PCR analysis of mRNA levels of adipogenic, lipogenic, and lipolytic genes in iWAT. **j** Real-time PCR analysis of mRNA levels of brown/mitochondria/β oxidation markers in BAT.

Table legends for Supplementary data 2 (Raw data for supplementary figures):

Raw data for Supplementary Fig. 3 (Protein levels of ChREBP and SCD1 in WT and *ATF3*$^{-/-}$ mice with or without restoration of ATF3 expression by adeno-associated virus containing ATF3 and GFP as control).

Raw data for Supplementary Fig. 4 (Positive correlations between ATF3 and HSL and CIDEA were validated in adipose tissue of patients by using Gene Expression Omnibus (GEO) and the GDS3679 dataset). **a** Positive correlations between ATF3 and HSL. **b** Positive correlations between ATF3 and CIDEA.

Raw data for Supplementary Fig. 5 (Overexpression of ATF3 decreased oil droplet deposition in 3T3-Ll cells after 8 days of differentiation). **b** Relative quantification of adipocyte differentiation.

Raw data for Supplementary Fig. 7 (Physiological effects of ATF3 inducer, ST32da, on mouse heart, kidney and liver). **a** Serum levels of BUN, creatinine, glucose, TG. **b** Serum level of GOT and GPT. **c** Liver weight.

Raw data for Supplementary Fig. 8 (The effect of ATF3 inducer, ST32da, in HFD-induced obese *ATF3*$^{-/-}$ mice). **a** Body weight (g). **b** food intake (g/mouse/day). **c** GTT. **d** ITT. **e** adipose tissue weight. **f** ChREBP and SCD1 gene expression. **g** Liver weight. **h** adipocyte size, adipocyte number per mm^2.

Raw data for Supplementary Fig. 9 (The effect of ATF3 inducer, ST32da, on serum adiponectin levels in HFD-fed *ATF3*$^{-/-}$ mice).

Raw data for Supplementary Fig. 10 (ATF3 inducer, ST32da, inhibited adipogenesis and induced browning in human primary adipocytes). **b** Gene expression levels of adipogenic, lipogenic, lipolytic, BAT, beta oxidation genes (Day 7). **c** Gene expression levels of adipogenic, lipogenic, lipolytic, BAT, beta oxidation genes (Day 14).

Table legends for Supplementary data 3:

The lists of Primers shown in Supplementary Table 2. Primers 1, Supplementary Table 3. Primers 2, Supplementary Table 4. Primers for ChIP assay.
