# Supplementary material for: Adipocyte browning and resistance to obesity in mice is induced by expression of ATF3
Source: Commun Biol. 2019 Oct 24;2:389. doi: 10.1038/s42003-019-0624-y (PMC6813364; doi:10.1038/s42003-019-0624-y)
Supplement: Supplementary file 6 — Reporting Summary [file 42003_2019_624_MOESM6_ESM.pdf]

nature research

Corresponding author(s): Heng Lin (COMMSBIO-18-1397A)

Last updated by author(s): Jul 9, 2019

# Reporting Summary

Nature Research wishes to improve the reproducibility of the work that we publish. This form provides structure for consistency and transparency in reporting. For further information on Nature Research policies, see Authors & Referees and the Editorial Policy Checklist .

## Statistics

For all statistical analyses, confirm that the following items are present in the figure legend, table legend, main text, or Methods section.

| n/a | Confirmed | |
|---|---|---|
| ☐ | ☒ | The exact sample size (*n*) for each experimental group/condition, given as a discrete number and unit of measurement |
| ☐ | ☒ | A statement on whether measurements were taken from distinct samples or whether the same sample was measured repeatedly |
| ☐ | ☒ | The statistical test(s) used AND whether they are one- or two-sided<br>*Only common tests should be described solely by name; describe more complex techniques in the Methods section.* |
| ☒ | ☐ | A description of all covariates tested |
| ☒ | ☐ | A description of any assumptions or corrections, such as tests of normality and adjustment for multiple comparisons |
| ☒ | ☐ | A full description of the statistical parameters including central tendency (e.g. means) or other basic estimates (e.g. regression coefficient) AND variation (e.g. standard deviation) or associated estimates of uncertainty (e.g. confidence intervals) |
| ☒ | ☐ | For null hypothesis testing, the test statistic (e.g. $F$, $t$, $r$) with confidence intervals, effect sizes, degrees of freedom and $P$ value noted<br>*Give P values as exact values whenever suitable.* |
| ☒ | ☐ | For Bayesian analysis, information on the choice of priors and Markov chain Monte Carlo settings |
| ☒ | ☐ | For hierarchical and complex designs, identification of the appropriate level for tests and full reporting of outcomes |
| ☒ | ☐ | Estimates of effect sizes (e.g. Cohen's $d$, Pearson's $r$), indicating how they were calculated |

*Our web collection on statistics for biologists contains articles on many of the points above.*

## Software and code

Policy information about availability of computer code

| | |
|---|---|
| Data collection | No software was used. |
| Data analysis | No software was used. |

For manuscripts utilizing custom algorithms or software that are central to the research but not yet described in published literature, software must be made available to editors/reviewers. We strongly encourage code deposition in a community repository (e.g. GitHub). See the Nature Research guidelines for submitting code & software for further information.

## Data

Policy information about availability of data

All manuscripts must include a data availability statement. This statement should provide the following information, where applicable:

- Accession codes, unique identifiers, or web links for publicly available datasets
- A list of figures that have associated raw data
- A description of any restrictions on data availability

All data generated or analysed during this study are included in this published article (and its Supplementary Information files).

# Field-specific reporting

Please select the one below that is the best fit for your research. If you are not sure, read the appropriate sections before making your selection.

☒ Life sciences ☐ Behavioural & social sciences ☐ Ecological, evolutionary & environmental sciences

For a reference copy of the document with all sections, see nature.com/documents/nr-reporting-summary-flat.pdf

# Life sciences study design

All studies must disclose on these points even when the disclosure is negative.

| | |
|---|---|
| Sample size | n = 3-10 in each experiments. |
| Data exclusions | No data were excluded from the analysis. |
| Replication | Each experiments were repeated more than 3 times to verify the reproducibility. |
| Randomization | There was no randomization for we used ATF3 knock out and wild type mice in this study. |
| Blinding | There was no blinding during data collection. |

# Reporting for specific materials, systems and methods

We require information from authors about some types of materials, experimental systems and methods used in many studies. Here, indicate whether each material, system or method listed is relevant to your study. If you are not sure if a list item applies to your research, read the appropriate section before selecting a response.

## Materials & experimental systems

| n/a | Involved in the study | |
|---|---|---|
| ☐ | ☒ | Antibodies |
| ☐ | ☒ | Eukaryotic cell lines |
| ☒ | ☐ | Palaeontology |
| ☐ | ☒ | Animals and other organisms |
| ☒ | ☐ | Human research participants |
| ☒ | ☐ | Clinical data |

## Methods

| n/a | Involved in the study | |
|---|---|---|
| ☒ | ☐ | ChIP-seq |
| ☒ | ☐ | Flow cytometry |
| ☒ | ☐ | MRI-based neuroimaging |

## Antibodies

| | |
|---|---|
| Antibodies used | The following antibodies were used: anti-ATF3 (cat# sc-188; 1:500; Santa Cruz Biotechnology), anti-UCP1 (cat# ab10983; 1:1000; Abcam), anti-ChREBP (cat# 81958; 1:1000; Abcam), anti-SCD1 (cat# 19862; 1:1000; Abcam), anti-adiponectin (cat# ab22554; 1:1000; Abcam), anti-actin (cat# sc-47778; Santa Cruz Biotechnology), anti-GAPDH (cat# ab8245; 1:10000; Abcam). |
| Validation | The validation of antibodies used in this study were statement in manufacturer's website. |

## Eukaryotic cell lines

Policy information about cell lines

| | |
|---|---|
| Cell line source(s) | 3T3-L1 cells and human pre-adipocyte cells were used in this study |
| Authentication | Both 3T3-L1 cells and human pre-adipocyte cells can be differentiated into mature adipocytes which containing oil droplet. |
| Mycoplasma contamination | The cell lines were not tested for Mycoplasma contamination. |
| Commonly misidentified lines (See ICLAC register) | There was no commonly misidentified cell lines used in this study. |

## Animals and other organisms

Policy information about studies involving animals; ARRIVE guidelines recommended for reporting animal research

| | |
|---|---|
| Laboratory animals | ATF3–/– C57BL/6 mice, 4~6 week-old were fed with high fat diet and ST32da (i.p. or oral ) for 12~16 weeks |
| Wild animals | C57BL/6 mice, 4~6 week-old were fed with high fat diet and ST32da (i.p. or oral ) for 12~16 weeks |

| | |
|---|---|
| Field-collected samples | The mice were housed in animal centers, 3-5 per cage, with a temperature of 18 to 26 degrees Celsius, humidity of 30 to 70 %, and a darkness of 12 hours. 8:00am~20:00pm light, 20:00~8:00 night .<br>For testing resistance to cold exposure, mice were individually caged and exposed to 4°C with free access to water. Core body temperature was monitored by using a rectal thermometer (KN-91, NATSUME) at the beginning and every 2 h after the start of cold exposure. Images were taken after cold exposure with use of an infrared thermographic camera (F30s, NEC Avio Infrared Technologies, Tokyo) |
| Ethics oversight | All procedures were performed according to the protocols approved by the Institutional Animal Care and Utilization Committee, Academia Sinica, Taipei, Taiwan. |

Note that full information on the approval of the study protocol must also be provided in the manuscript.
